# Phosphoproteomics of collagen receptor networks reveals SHP-2 phosphorylation downstream of wild-type DDR2 and its lung cancer mutants

**DOI:** 10.1042/BJ20121750

**Published:** 2013-08-29

**Authors:** Leo K. Iwai, Leo S. Payne, Maciej T. Luczynski, Francis Chang, Huifang Xu, Ryan W. Clinton, Angela Paul, Edward A. Esposito, Scott Gridley, Birgit Leitinger, Kristen M. Naegle, Paul H. Huang

**Affiliations:** *Protein Networks Team, Division of Cancer Biology, Institute of Cancer Research, London SW3 6JB, U.K.; †National Heart and Lung Institute, Imperial College London, London SW7 2AZ, U.K.; ‡Blue Sky Biotech Inc., Worcester, MA 01605, U.S.A.; §Cancer Research UK Tumour Cell Signalling Unit, Institute of Cancer Research, London SW3 6JB, U.K.; ∥Department of Biomedical Engineering, Washington University in St. Louis, St. Louis, MO 63130, U.S.A.

**Keywords:** cell signalling, collagen, discoidin domain receptor, lung cancer, mass spectrometry, phosphoproteomics, CDK1, cyclin-dependent kinase 1, DDR, discoidin domain receptor, DMEM, Dulbecco’s modified Eagle’s medium, DYRK1A, dual-specificity tyrosine-phosphorylation-regulated kinase 1A, EGFR, epidermal growth factor receptor, ERK, extracellular-signal-regulated kinase, EV, empty vector, GO, Gene Ontology, HEK, human embryonic kidney, HRP, horseradish peroxidase, IL, interleukin, IMAC, immobilized metal-ion-affinity chromatography, KD, kinase domain, mAb, monoclonal antibody, MCAM, multiple clustering analysis methodology, NCK1, non-catalytic region of tyrosine kinase adaptor protein 1, PIK3C2A, phosphatidylinositol-4-phosphate 3-kinase, catalytic subunit type 2α, PLCL2, phospholipase C-like 2, RFB, radiometric filter binding, RTK, receptor tyrosine kinase, SCC, squamous cell carcinoma, SHIP-2, SH2 (Src homology 2)-domain-containing inositol phosphatase 2, SHP-2, Src homology 2 domain-containing protein tyrosine phosphatase 2, SRM, selective reaction monitoring, TDA, template-directed assembly, TEAB, triethylammonium bicarbonate, TFA, trifluoroacetic acid

## Abstract

Collagen is an important extracellular matrix component that directs many fundamental cellular processes including differentiation, proliferation and motility. The signalling networks driving these processes are propagated by collagen receptors such as the β1 integrins and the DDRs (discoidin domain receptors). To gain an insight into the molecular mechanisms of collagen receptor signalling, we have performed a quantitative analysis of the phosphorylation networks downstream of collagen activation of integrins and DDR2. Temporal analysis over seven time points identified 424 phosphorylated proteins. Distinct DDR2 tyrosine phosphorylation sites displayed unique temporal activation profiles in agreement with *in vitro* kinase data. Multiple clustering analysis of the phosphoproteomic data revealed several DDR2 candidate downstream signalling nodes, including SHP-2 (Src homology 2 domain-containing protein tyrosine phosphatase 2), NCK1 (non-catalytic region of tyrosine kinase adaptor protein 1), LYN, SHIP-2 [SH2 (Src homology 2)-domain-containing inositol phosphatase 2], PIK3C2A (phosphatidylinositol-4-phosphate 3-kinase, catalytic subunit type 2α) and PLCL2 (phospholipase C-like 2). Biochemical validation showed that SHP-2 tyrosine phosphorylation is dependent on DDR2 kinase activity. Targeted proteomic profiling of a panel of lung SCC (squamous cell carcinoma) DDR2 mutants demonstrated that SHP-2 is tyrosine-phosphorylated by the L63V and G505S mutants. In contrast, the I638F kinase domain mutant exhibited diminished DDR2 and SHP-2 tyrosine phosphorylation levels which have an inverse relationship with clonogenic potential. Taken together, the results of the present study indicate that SHP-2 is a key signalling node downstream of the DDR2 receptor which may have therapeutic implications in a subset of DDR2 mutations recently uncovered in genome-wide lung SCC sequencing screens.

## Abstract 2

The present study characterizes integrin and DDR2 signalling networks activated by collagen. Using clustering approaches, DDR2-specific signalling components such as SHP-2 were identified. We further demonstrate that SHP-2 is phosphorylated by a subset of DDR2 lung cancer mutants.

## INTRODUCTION

Collagen is the most abundant protein in mammals and plays a critical role in maintaining tissue structural integrity [1]. There is an increasing appreciation that collagen has additional functions in propagating signal transduction networks which drive cellular behaviour in both physiological and pathological conditions. A number of different transmembrane collagen receptors have been identified, with the integrin family of receptors being the most well-studied [2]. Integrins are heterodimeric type 1 transmembrane molecules composed of an α and β subunit that engage a wide variety of extracellular matrix components and initiate intracellular signalling networks through the recruitment of downstream effector proteins [3,4]. The primary collagen-binding integrins are the α1β1 and α2β1 heterodimers, and these receptors have been shown to bind specific consensus sequence motifs in triple-helical regions on fibrillar collagen molecules [2,5]. In addition to the integrins, the DDRs (discoidin domain receptors) are another class of collagen-binding receptors. They differ from the integrins in that they belong to the RTK (receptor tyrosine kinase) superfamily and have intrinsic phosphotransferase activity through their cytoplasmic KD (kinase domain) [6–8]. Although classical growth factor-activated RTKs, such as the EGFR (epidermal growth factor receptor), display rapid and transient receptor activation dynamics [9], the DDRs are unique in that they exhibit delayed and sustained receptor phosphorylation upon binding to collagen [6,7]. The use of synthetic peptide toolkits to probe for receptor-binding specificity indicates that the DDRs bind to specific amino acid consensus sequences on fibrillar collagen that are distinct from integrin-binding motifs [10–12].

DDR2 is one of two members of the DDR family. It is highly expressed in mesenchymal cells and is important for a variety of developmental processes, in particular bone and cartilage formation [13]. DDR2 binds exclusively to fibrillar collagen and collagen X [14]. DDR2 also contributes to disease progression, including hepatic fibrosis, osteoarthritis and cancer [15–18]. Large-scale phosphoproteomic screens of human tumours have identified DDR2 to be highly phosphorylated in a subset of non-small-cell lung cancers, cholangiocarcinomas and sarcomas [15,17,19]. Furthermore, cancer genome sequencing efforts have identified a series of oncogenic DDR2 point mutations that occur at low frequency in lung SCC (squamous cell carcinoma) [20,21]. A recent RNAi screen of protein tyrosine kinases has revealed that DDR2 is among a group of kinases that is required for extracellular matrix rigidity sensing and polarization in fibroblasts, implicating a role in mechanotransduction [22]. Although some of the functional roles of DDR2 in human health and disease have been identified, the signalling networks driving these biological processes remain largely unknown.

In the present study, we have performed an unbiased quantitative MS-based phosphoproteomic analysis of collagen receptor signalling networks upon stimulation with collagen I. Temporal analysis over seven time points identified 424 phosphorylated proteins. Using MCAM (multiple clustering analysis methodology), we find that a subset of phosphorylation sites on important signalling proteins such as SHP-2 (Src homology 2 domain-containing protein tyrosine phosphatase 2), NCK1 (non-catalytic region of tyrosine kinase adaptor protein 1), LYN, SHIP-2 [SH2 (Src homology 2)-domain-containing inositol phosphatase 2], PLCL2 (phospholipase C-like 2) and PIK3C2A (phosphatidylinositol-4-phosphate 3-kinase, catalytic subunit type 2α) strongly cluster with DDR2 phosphorylation dynamics, implicating these proteins as candidate downstream effectors of DDR2 signalling. A survey of lung SCC mutants of DDR2 using targeted proteomics show that SHP-2 is tyrosine-phosphorylated in a subset of these mutants. The present study demonstrates that SHP-2 is a key downstream component of DDR2 signalling and highlights the utility of phosphoproteomics in providing new insights into cell–matrix signalling.

## EXPERIMENTAL

### Cell culture, transfection and selection of cells

HEK (human embryonic kidney)-293 cells were obtained from the A.T.C.C. and cultured in DMEM (Dulbecco's modified Eagle's medium) with 10% FBS, 2 mM glutamine, 100 units/ml penicillin and 100 mg/ml streptomycin in an atmosphere of 95% air/5% CO_2_ at 37°C. For expression of DDR2, HEK-293 cells were transfected with pcDNA3.1-DDR2 [11,23] or an empty vector control using the calcium phosphate method and selected in 400 μg/ml zeocin (Invivogen) as described previously [24,25]. For DDR2 mutants, the QuikChange® XL site-directed mutagenesis kit (Stratagene) was used according to the manufacturer's protocol. Primers used for the mutagenesis are detailed in Supplementary Table S1 (at http://www.biochemj.org/bj/454/bj4540501add.htm. Selected cells were pooled and analysed for DDR2 expression by immunoblotting analysis.

### Immunoblotting

HEK-293-DDR2 or mutant cells were stimulated with 20 μg/ml acetic-acid-solubilized rat tail collagen I (Sigma) at the indicated time points and lysed with RIPA lysis buffer [25 mM Tris/HCl (pH 7.6), 150 mM NaCl, 1% Nonidet P40, 1% sodium deoxycholate and 0.1% SDS] supplemented with protease and phosphatase inhibitors (Thermo Pierce) at 4°C. Equal amounts of protein, as determined by the bicinchroninic acid protein assay (Thermo Pierce), was applied in each lane of 4–12% or 10% Bis-Tris gels (Invitrogen). Following one-dimensional separation and transfer on to PVDF membrane, the membrane was incubated overnight at 4°C with 1:1000 dilutions of goat anti-DDR2 (R&D Systems), mouse anti-phosphotyrosine [4G10 (Millipore) or PY100 (Cell Signaling Technology)] or rabbit anti-SHP2 (Santa Cruz Biotechnology) antibodies, a 1:500 dilution of a rabbit anti-(SHP2 p-Tyr^542^) (Cell Signaling Technology) antibody or with a 1:5000 dilution of a mouse anti-α-tubulin (Sigma). After a 1 h incubation with secondary antibodies [HRP (horseradish peroxidase)-conjugated anti-goat IgG, anti-rabbit IgG or anti-mouse IgG at a 1:10000 dilution (Stratech Scientific)], immunoreactive bands were visualized by enhanced chemiluminescence (pico-L; Thermo Pierce) and the blots were exposed to X-ray XAR film (Kodak).

### Flow cytometry

HEK-293-DDR2 cells were grown in six-well plates for 24 h. Cells were then dissociated with non-enzymatic cell dissociation solution (Sigma) and resuspended in PBS containing 1% BSA. The cells were incubated for 30 min on ice with primary mAbs (monoclonal antibodies) at 10 μg/ml in 100 μl of PBS/BSA. Cells were then washed three times with PBS/BSA and incubated with FITC-conjugated goat anti-mouse IgG (F-9006, Sigma) for 30 min on ice. After three washes as above, the cells were resuspended in 2% formaldehyde in PBS. Data were subsequently collected on a BD LSRFortessa cell analyser using BD FACSDiva software 6.0 (BD Biosciences), and further analysed on FlowJo software 7.6.4 (Tree Star). Mouse anti-(integrin α1) mAb, clone FB12, was purchased from Millipore Chemicon and mouse anti-(integrin α2) mAb, clone AK7, was from AbD Serotec. Mouse anti-(integrin β1), clone P5D2, was purified from hybridoma cells obtained from the Developmental Studies Hybridoma Bank, University of Iowa, Iowa City, IA, U.S.A.

### Sample preparation and phosphopeptide immunoprecipitation

HEK-293-DDR2 cells were washed with PBS and incubated overnight in serum-free media prior to stimulation with 20 μg/ml acetic-acid-solubilized rat tail collagen I (Sigma) at the time points indicated. Cells were then lysed in 8 M urea, and subjected to reduction, alkylation and trypsin digestion as described previously [26]. Briefly, samples were reduced with 10 mM DTT for 1 h at 56°C and alkylated with 55 mM iodoacetamide for 1 h at room temperature (20°C). Samples were digested with 40 μg of trypsin (Promega) overnight at room temperature. Peptides were desalted on a C_18_ Sep-Pak Plus cartridge (Waters), eluted with 25% acetonitrile and freeze-dried to dryness. Freeze-dried peptides were subjected to labelling with the iTRAQ 8-plex reagent (ABSciex) according to the manufacturer's instructions [27]. Enrichment of phosphotyrosine peptides was achieved using peptide immunoprecipitation as described previously [26,28]. Briefly, 30 μl of Protein G Plus-agarose beads (Sigma) were incubated with 15 μg of each of the anti-phosphotyrosine antibodies, pY100 (Cell Signaling Technology) and 4G10 (Millipore), in 200 μl of immunoprecipitation buffer [100 mM Tris, 100 mM NaCl and 1% Nonidet P40 (pH 7.4)] for 8 h at 4°C. Beads were washed with rinse buffer [100 mM Tris and 100 mM NaCl (pH 7.4)] and retained peptides were eluted from the antibody with 70 μl of elution buffer [100 mM glycine (pH 2.5)] for 30 min at room temperature. The eluate from the immunoprecipitation was subjected to IMAC (immobilized metal-ion-affinity chromatography) enrichment before MS analysis. After the depletion of phosphotyrosine-containing peptides, 200 μg of the supernatant from the immunoprecipitation was subjected to two rounds of IMAC for global phosphorylation analysis.

### IMAC phosphopeptide purification and LC-MS/MS analysis

IMAC enrichment of phosphorylated peptides was performed using Ni-NTA (Ni^2+^-nitrilotriacetate) agarose beads (Qiagen) using a protocol adapted from [29]. A volume of 100 μl (for phosphotyrosine peptides) or 200 μl (for global phosphopeptide enrichment) of beads were washed three times with water, and incubated with 100 mM EDTA (pH 8.0) for 30 min. The beads were then washed three times with water, and incubated with 100 mM FeCl_3_ for 45 min. After removing excess metal ions, beads were washed three times with water. The iTRAQ-labelled eluent from the immunoprecipitation was acidified with 25 μl of 10% TFA (trifluoroacetic acid) and added to the iron-chelated resin. Then 200 μl of 80% acetonitrile and 0.1% TFA was added to the mixture before incubation for 1 h at room temperature. Beads were washed twice with 80% acetonitrile and 0.1% TFA, followed by two washes with 80% acetonitrile and 0.1% acetic acid, and two washes of 0.1% acetic acid. For the global phosphorylation analysis, the flowthrough from the first IMAC was subjected to an additional round of IMAC enrichment. The beads from the three IMAC enrichments (one for the phosphotyrosine immunoprecipitation and two for the global enrichment) were separately eluted with either 40 μl (phosphotyrosine peptides) or 80 μl (global phosphopeptide enrichment) of 250 mM sodium phosphate (pH 8.0) for 30 min and loaded directly on to a reverse-phase (C_18_) pre-column (100 μm internal diameter, packed with 10 cm of 10 μm C_18_ beads). The pre-column was attached to an analytical column (50 μm internal diameter fused silica capillary packed with 10 cm of 5 μm C_18_ beads) with an integrated electrospray bottleneck tip with an approximate 1 μm orifice. For the phosphotyrosine analysis, peptides were eluted using a 125-min gradient with solvents A (1% acetic acid) and B (water/acetonitrile/acetic acid at 10:89:1, by vol.): 10 min from 0 to 13% B, 95 min from 13 to 42% B, 10 min from 42 to 60% B, and 10 min from 60 to 100% B. For the global phosphopeptide analysis, peptides were eluted using a 220-min gradient with: 10 min from 0 to 13% B, 190 min from 13 to 42% B, 10 min from 42 to 60% B, and 10 min from 60 to 100% B. Eluted peptides were directly electrosprayed into a QqTOF mass spectrometer (QSTAR Elite; ABSciex) operated in information-dependent acquisition mode. MS/MS spectra of the five most intense peaks with two to five positive charges in the MS scan were automatically acquired with previously selected peaks excluded for 60 (phosphotyrosine) or 90 (global phosphorylation) s.

### Data analysis

MS/MS spectra were searched against a *Homo sapiens* protein database (NCBI) by using MASCOT (version 2.2; Matrix Science) with trypsin as the enzyme and allowing up to three missed cleavages. Oxidation of methionine and phosphorylation of serine, threonine, tyrosine were included as variable modifications (0.15 Da MS/MS tolerance and 2.2 Da peptide tolerance), while carbamidomethylation of cysteine and iTRAQ modification of the -NH_2_ lysine side chain and the N-terminus were included as fixed modifications. Peptide sequence validation was further confirmed manually for each of the peptides identified by checking the raw MS/MS data for possible mixed spectra, non-assigned abundant peaks and phosphorylation position. Phosphopeptide quantification was determined via Protein Pilot (ABSciex) by calculating the peak area for iTRAQ marker ions. The Protein Pilot software corrects for isotopic contamination associated with iTRAQ reagents as the signal for each isotopic tag contributes to the signal of the other tags. Quantification results were additionally manually validated. Each condition was normalized against the 121.1 channel to obtain fold changes across all seven conditions. To account for protein loading differences in the seven samples, a small fraction (~0.1%) of the supernatant from the tyrosine phosphopeptide immunoprecipitation was analysed by LC-MS/MS, thereby providing quantification for the non-phosphorylated peptides in each sample. Protein loading quantification was then used to normalize the iTRAQ marker ion data for phosphorylated peptides.

### SRM (selective reaction monitoring)

For SRM assays, cell lysates were prepared as detailed above for iTRAQ experiments. Analyses were performed using the equivalent of the same amount of cell lysate (1.5–2.4 mg depending on experiment) per condition. Following digestion and Sep-Pak desalting, phosphotyrosine-containing peptides were immunoprecipitated using 10 μg of the pY100 antibody and 30 μl of Protein G Plus-agarose beads (Calbiochem). Immunoprecipitated peptides were eluted in 40 μl of elution buffer [100 mM glycine (pH 2.5)] and beads were removed by centrifugation at 5000 ***g*** for 3 min. Eluted peptides were then transferred to a fresh tube, and 2 μl of a heavy peptide standard mix was added per sample to allow for normalization of precipitated endogenous peptide levels between runs. Heavy peptides sequences are detailed in Supplementary Table S2 (at http://www.biochemj.org/bj/454/bj4540501add.htm).

Samples were analysed using a Q-Trap 4000 instrument (ABSciex). Samples containing heavy peptide standards were loaded on to a reverse-phase (C_18_) pre-column (100 μm internal diameter, packed with 5–10 cm of 10 μm C_18_ beads). The pre-column was attached to an analytical column (50 μm internal diameter fused silica capillary packed with 10 cm of 5 μm C_18_ beads) with an integrated electrospray bottleneck tip with an approximate 1 μm orifice. Peptides were eluted using a 75-min gradient with solvent A (1% acetic acid) and B (water/acetonitrile/acetic acid at 10:89:1, by vol.): 10 min from 0 to 10% B, 45 min from 10 to 34% B, 10 min from 34 to 47% B, and 10 min from 47 to 100% B. Transitions were monitored for endogenous and heavy phosphopeptides as detailed in Supplementary Table S3 (at http://www.biochemj.org/bj/454/bj4540501add.htm). Peak areas were calculated using Analyst v1.5 software, and peak areas for endogenous peptide transitions were normalized to the corresponding transitions for the spiked heavy peptide standards.

### Dataset preparation and MCAM implementation

The PTMScout [30] interface was used to analyse the phosphoproteomic dataset. The parameters of clustering used in the initial MCAM round and those pruned from the final round are provided in Supplementary Table S4 (at http://www.biochemj.org/bj/454/bj4540501add.htm). Set pruning was determined by removing those sets whose removal improved the overall biological enrichment by 10% or greater, while not decreasing the impact of any one biological category by more than 5%. Statistical significance of biological enrichment was calculated using the PTMScout MCAM interface for GO (Gene Ontology) terms, Pfam domains, Scansite binding and kinase predictions, linear amino acid sequence motifs, and enrichment for the domain a phosphorylation site falls in. This analysis used the default PTMScout parameters of 5×10^−2^ for an α-corrected value, corrected by the Hochberg and Benjamini FDR procedure [31], 1×10^−5^ for significant Pfam domain consideration, 1×10^−2^ cut-off for motif search analysis, and a Scansite cut-off of 3. At the time statistical significance of enrichment was calculated, GO terms on PTMScout were at version 1.2 and downloaded June 26, 2012 from GO (http://www.geneontology.org). The Matlab code for enrichment analysis and pruning was downloaded from http://ptmscout.mit.edu/MCAM_July_2011.zip. Further details regarding the process of MCAM, including enrichment analysis and pruning, can be found in [32].

### *In vitro* kinase assay

Using a TDA (template-directed assembly) approach which organizes functional complexes of membrane-associated proteins, His-tagged DDR2 and/or Src (see Supplementary Figure S1C at http://www.biochemj.org/bj/454/bj4540501add.htm for sequences) were mixed at the indicated amounts with a 1:5000 ratio of TDA/enzyme. Enzyme solutions (2×; 1.5 μM) were prepared in assay buffer 1 [AB1; 20 mM Hepes (pH 7.2), 10 mM MgCl_2_ and 0.01% Tween 20] and mixed with equal volumes of either (i) 200 μM ATP and 200 μM Axltide peptide substrate (KKSRGDYMTMQIG) in AB1 for loading into PAGE gels, or (ii) 200 μM ATP (with 1.5 μCi of [γ-^32^P]ATP) and 200 μM peptide substrate in AB1 for RFB (radiometric filter binding) assays. Each activity assay was incubated for 30 min at room temperature. RFB assay samples were stopped by spotting 20 μl on to P-81 phosphocellulose filters pre-wetted with 0.75% phosphoric acid. Filters were washed four times with 35 ml of 0.75% phosphoric acid, then immersed in 3 ml of scintillant (Ultima gold; PerkinElmer) and counted on a Beckman LSC-1800 liquid scintillation counter.

Gel assay samples were stopped by the addition of reducing/denaturing loading buffer, then boiled for 10 min, quickly cooled, and loaded into 30 μl wells on a 10% precast Tris-glycine gel (Bio-Rad Laboratories). Gels were run, stained with Coomassie Brilliant Blue and destained. Excised gel bands were diced and destained in consecutive 50% acetonitrile/50 mM TEAB (triethylammonium bicarbonate) and 50 mM TEAB washes until all of the stain was removed. The gel pieces were shrunk amd then reduced with 10 mM DTT and alkylated with 55 mM iodoacetamide. The proteins were subjected to digestion with modified porcine trypsin (Promega) and incubated at 37°C overnight. After digestion, the proteolytic peptides were extracted with 50% acetonitrile/formic acid and TEAB washes, the extract solution was reduced in volume to approximately 10 μl in a speed-vac and subjected to LC-MS/MS sequencing analysis.

### ELISA

Cell lines were serum-starved overnight before stimulation with collagen I (Sigma) or acid control at the time points indicated and lysed. For P5D2-blocking experiments, HEK-293-DDR2 cells were pre-treated with P5D2 (10 μg/ml, R&D Systems) for 30 min before stimulation with collagen for 24 h and lysis. Lysis buffer for extraction of DDR2, PLCL2, SHIP2 and SHP-2 was 1% Nonidet P40, 20 mM Tris (pH 8.0), 137 mM NaCl, 10% glycerol and 4.5 mM EDTA; for LYN lysis buffer was 1 mM EDTA, 0.5% Triton X-100 and 6 M urea in PBS (pH 7.4); and for NCK1 lysis buffer was 50 mM Hepes, 0.1 mM EDTA, 0.1 mM EGTA, 120 mM NaCl and 0.5% Nonidet P40 (pH 7.5). For determination of phospho-SHP2 Tyr^542^ levels, cells were lysed in 50 mM Hepes, 5 mM EDTA, 0.1 mM EGTA, 0.5% Nonidet P40 and 120 mM NaCl (pH 7.5). All lysis buffers were supplemented with protease and phosphatase inhibitors (Thermo Pierce). An ELISA for phospho-DDR2 was performed using the Human Phospho-DDR2 DuoSet IC ELISA kit (R&D Systems). Phospho-SHP2 was determined using the Phospho-SHP2 (Y542) DuoSet IC ELISA kit (R&D Systems). All of the remaining phosphoproteins were measured by custom sandwich ELISAs using the following capture antibodies diluted in PBS: total SHP-2 capture antibody (2 μg/ml) from the SHP-2 DuoSet IC kit (R&D Systems); anti-PLCL2 mAb clone 2D10 (5 μg/ml, Sigma); anti-LYN polyclonal antibody (5 μg/ml, catalogue number ab77400, Abcam); anti-SHIP2 mAb, clone T.194.8 (1:500 dilution, Thermo Scientific); and anti-NCK1 mAb, clone 714506 (3 μg/ml, R&D Systems). ELISA plates (Costar) were coated with capture antibody overnight at room temperature and subsequently blocked with 1% BSA in PBS for 2 h at room temperature.

Each sample (100 μl) was loaded in duplicate and incubated for 2 h at room temperature, followed by the addition of 100 μl of HRP-conjugated phosphotyrosine mouse mAb (1:1000 dilution, pY100, Cell Signaling Technology) in diluent [20 mM Tris, 137 mM NaCl, 0.05% Tween 20 and 0.1% BSA (pH 7.4)] (2 h at room temperature). The reaction was visualized by the addition of 100 μl of chromogenic substrate [TMB (3,3′,5,5′-tetramethylbenzidine), R&D Systems] for 30 min and the reaction was stopped with 50 μl of 1 M H_2_SO_4_. Absorbance at 450 nm was measured using an ELISA plate reader. Plates were washed five times with wash buffer [PBS (pH 7.4) containing 0.1% Tween 20] after each step.

### Colony formation assay

Cell lines were resuspended in a solution composed of 1 mg/ml rat tail collagen (BD Biosciences) in DMEM with the pH adjusted to 7.4 with 1 M NaOH. Cells (400) were seeded in each well of a 96-well plate in 100 μl of the collagen solution. After the collagen gel solidified, 100 μl of DMEM (10% FBS) was added to the top of the gel and media was changed every other day. Cells were grown for 15 days and colonies were counted.

## RESULTS

### Generation of cell line and MS experimental strategy

**Figure 1:**
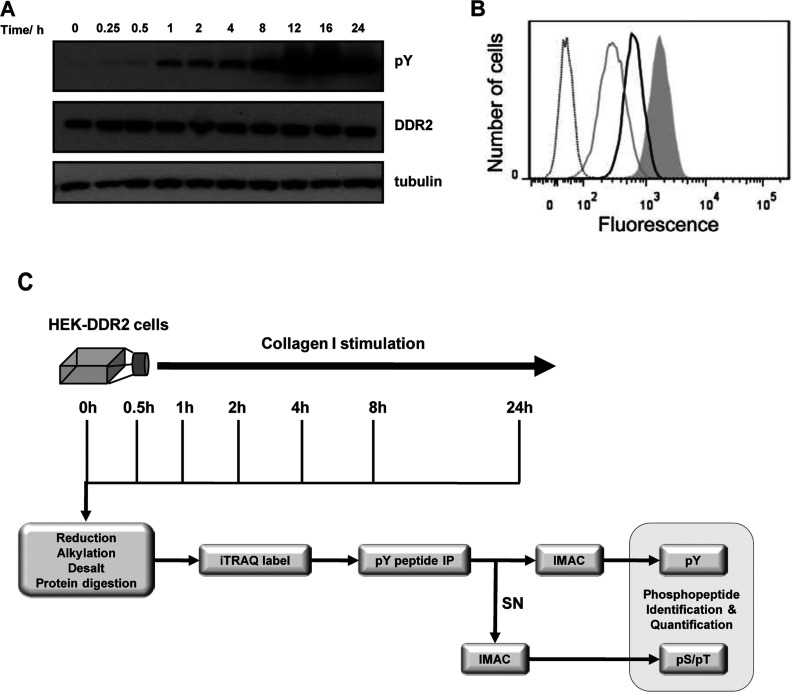
Characterization of cell line and MS experimental strategy (**A**) Immunoblot of DDR2 activation over 24 h after stimulation with 20 μg/ml collagen I. Phosphotyrosine (pY) is measured by the 4G10 antibody. (**B**) Integrin expression in HEK-293-DDR2 cells. The cells were stained on ice with 10 μg/ml of the primary mAbs followed by FITC-conjugated goat anti-mouse IgG and analysis by flow cytometry. Dotted line, secondary antibody only; grey line, anti-(integrin α1); black line, anti-(integrin α2); filled grey histogram, anti-(integrin β1). Shown are representative data for three experiments. (**C**) Experimental strategy for phosphoproteomic analysis. HEK-293-DDR2 cells were stimulated across seven time points after the addition of collagen I and lysed. Proteins were extracted, digested and isotopically labelled with the iTRAQ 8-plex reagent. Phosphotyrosine enrichment was performed by anti-phosphotyrosine immunoprecipitation followed by IMAC. Global phosphorylation was assessed by two rounds of IMAC enrichment of the supernatant (SN) of the immunoprecipitation. Eluted peptides were then analysed by LC-MS/MS and the resulting data were processed for phosphopeptide identification and quantification as described in the Experimental section.

To identify the phosphorylation-mediated signalling networks downstream of DDR2 activation, HEK-293 cells were transduced to stably express DDR2 (HEK-293-DDR2 cells) which were then pooled before immunoblotting to assay for DDR2 expression. The HEK-293 cell line has been extensively used as a model system to investigate DDR signalling [6,11,33]. Stimulation of HEK-293-DDR2 cells with acid-soluble collagen I resulted in the activation of DDR2 with delayed and sustained tyrosine phosphorylation over 24 h (Figure 1A) as reported previously [6]. These cells maintained high expression levels of the major collagen integrins, β1, α1 and α2 integrin subunits, as demonstrated by flow cytometry assessment of surface expression levels of these receptors (Figure 1B). Consistent with a recent characterization of integrin levels in HEK-293 cells [34], HEK-293-DDR2 cells maintained high levels of β1 integrin subunit. Additionally, the cells expressed significant levels of the α2 subunit and lower levels of the α1 subunit.

In order to minimize any confounding signals that may be associated with growth factors commonly found in serum in cell culture media, the HEK-293-DDR2 cells were serum-starved before stimulation with 20 μg/ml collagen I over seven time points (0–24 h) (Figure 1C). Cells were lysed and the samples were prepared in two biological replicates as described in the Experimental section. Peptides from the seven conditions were stable isotope-labelled with the iTRAQ isobaric reagent, mixed and tyrosine-phosphorylated peptides were immunoprecipitated with a mixture of pan-specific anti-phosphotyrosine antibodies. Following immunoprecipitation, the phosphotyrosine peptides were further enriched using IMAC and analysed by LC-MS/MS. After the sample was depleted of phosphotyrosine-containing peptides, a global enrichment of phosphopeptides was performed with the iTRAQ-labelled supernatant from the immunoprecipitation being subjected to two rounds of IMAC before LC-MS/MS. In total, quantitative phosphorylation profiles were identified for 646 phosphosites on 649 phosphopeptides on 424 proteins across the seven time points (see the list of protein phosphorylation sites at http://www.biochemj.org/bj/454/bj4540501add.htm). Of the phosphopeptides that were identified, manual sequencing of mass spectra unambiguously localized the site of phosphorylation on 549 of the peptides.

### DDR2 receptor phosphorylation upon collagen I activation

**Figure 2:**
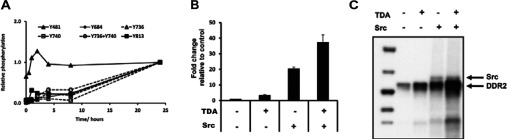
DDR2 receptor phosphorylation and *in vitro* kinase assay (**A**) Plots of relative DDR2 phosphorylation levels as a function of time after collagen stimulation. Temporal profiles of DDR2 sites show distinct responses to collagen stimulation. Measurements are expressed relative to the 24 h time point. (**B**) *In vitro* kinase assay measuring the incorporation of ^32^P into the Axltide substrate peptide. Equal amounts (750 nM) of Src and DDR2 were mixed together with 300 pM TDA in kinase assay buffer. Data represent the fold change relative to control (DDR2). (**C**) Immunoblot of total phosphotyrosine levels (using the 4G10 antibody) obtained from the *in vitro* kinase reaction showing increased phosphorylation levels that correlate with DDR2 kinase activity.

The phosphoproteomic analysis revealed six phosphorylated peptides containing five phosphosites on DDR2 that were dynamically regulated upon collagen activation (Figure 2A). Two of these sites (Tyr^736^ and Tyr^740^) are located on the activation loop of the receptor which contains three tyrosine residues (Tyr^736^/Tyr^740^/Tyr^741^) that are highly conserved among a number of RTKs, including the insulin receptor and c-Met [26,35]. These two sites occurred either as a singly or a doubly phosphorylated peptide (Tyr^736^+Tyr^740^) and displayed a sharp increase in phosphorylation levels from 8 to 24 h, which is in agreement with the delayed activation kinetics of this receptor [6]. Two additional novel phosphosites which have not been previously described and are found in the KD of the receptor, Tyr^684^ and Tyr^813^, exhibited similar kinetics, with maximal phosphorylation achieved at 24 h. In contrast, the Tyr^481^ site present on the juxtamembrane domain of the receptor displayed constitutive phosphorylation levels and had a distinct profile compared with the other receptor phosphorylation sites.

To confirm that the conserved tyrosine sites on the activation loop are associated with DDR2 kinase activity, we performed an *in vitro* kinase assay using TDA of His-tagged DDR2 in the presence of Src [36]. It has previously been shown that Src is required for activation of DDR2 in an *in vitro* setting and when co-expressed in baculovirus [37]. The TDA approach has been used to restore biological relevant functionality to recombinant membrane-associated proteins, or fragments thereof [38–40]. TDA of DDR2 in combination with Src enhanced both the kinase activity as well as the total tyrosine phosphorylation levels of the receptor compared with Src or TDA treatment alone (Figures 2B and 2C). Src itself does not phosphorylate the Axltide substrate used in these experiments (Supplementary Figure S1A). The resulting kinase assay reaction was run on a gel and the band corresponding to DDR2 (Supplementary Figure S1B) was excised and subjected to LC-MS/MS analysis to identify the phosphorylation sites on DDR2 that are present upon kinase activation (Table 1). The sequencing analysis confirmed that two sites (Tyr^740^ and Tyr^741^) on the activation loop were phosphorylated only upon the addition of Src and TDA at which maximal kinase activity was observed. In agreement with the cellular phosphoproteomic data, Tyr^481^ was seen to be phosphorylated under all experimental conditions, even in its basal inactive state, indicating constitutive phosphorylation of this site. Interestingly, a number of previously undescribed serine phosphorylation sites (Ser^446^, Ser^674^ and Ser^696^) were also identified in this *in vitro* analysis, and additional future work will be required to determine the functional relevance of these phosphosites in cells.

**Table 1:** List of phosphorylation sites identified in the *in vitro* kinase assay

		Condition				
Phosphosite	Sequence	DDR2	DDR2+TDA	DDR2+Src	DDR2+Src+TDA	DDR2 negative control
Ser^446^	RMLDDEMTVSLpSLPSDSSMFNNNR	**+**	**+**	**+**	**+**	
Ser^461^	SSpSPSEQGSNSTYDR	**+**	**+**	**+**	**+**	**+**
Tyr^471^	SSSPSEQGSNSTpYDR			**+**	**+**	**+**
Tyr^481^	IFPLRPDpYQEPSR	**+**	**+**	**+**	**+**	
Ser^674^	HEPPNpSSSDVR				**+**	
Ser^696^	FMATQIApSGMK	**+**	**+**			**+**
Tyr^740^	NLYSGDpYYR				**+**	
Tyr^741^	NLYSGDYpYR				**+**	

### Multiple clustering analysis identifies components of the DDR2 signalling network

**Figure 3:**
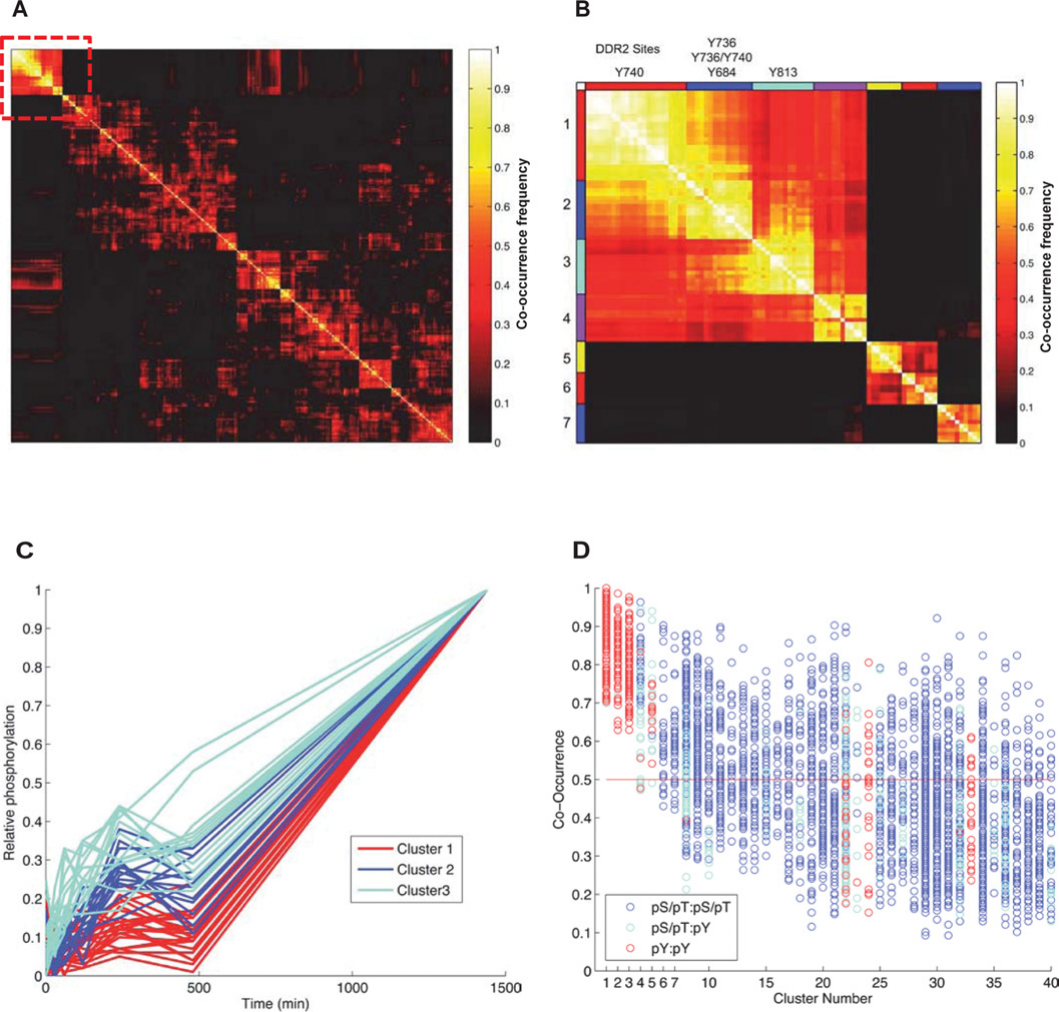
MCAM analysis of the phosphoproteomic dataset (**A**) Co-occurrence matrix for the phosphoproteomic dataset. Co-occurrences are calculated by counting the number of times any two phosphopeptides cluster together across all 216 clustering sets. The matrix is then normalized as a percentage of the number of times they cluster out of 216 times and subsequently clustered using hierarchical clustering with Ward linkage. The first seven clusters that display higher-than-average co-occurrence are highlighted by the red box. (**B**) The co-occurrence map of the first seven clusters expanded from (**A**). Cluster 1 contains DDR2 Tyr^740^ as well as SHP2 Tyr^62^. Cluster 2 contains three DDR2 phosphopeptides containing Tyr^736^, Tyr^736^/Tyr^740^ and Tyr^618^. Cluster 3 contains DDR2 Tyr^813^. (**C**) The dynamics of the members of each of the three DDR2 phosphorylation site-containing robust clusters. (**D**) Distribution of co-occurrences in the ensemble clustering result. A single hard clustering from the ensemble is obtained by cutting the Ward-linked hierarchically clustered co-occurrence matrix in (A) into 40 clusters. The co-occurrences within every cluster is then plotted to give an idea of the distribution. Comparison of phosphotyrosine/phosphotyrosine (pY/pY) sites are in red, phosphoserine/phosphothreonine (pS/pT):phosphoserine/phosphothreonine (pS/pT) are in blue, and cyan is of the co-occurrence events between phosphotyrosine (pY) and phosphoserine/phosphothreonine (pS/pT) sites.

Since our phosphoproteomic analysis represents the activation of both DDR2 and the integrins, we subjected the data to MCAM to isolate the DDR2-specific signalling components. MCAM is an unsupervised learning approach that requires no previous assumptions of the network properties under study and applies a variety of clustering algorithms, number of target clusters (K), distance metrics and data transformations, in a combinatorial fashion. Following application of clustering, feature selection is employed in the form of statistically enriched biological annotations within clusters [32]. The unambiguous phosphorylation dataset was chosen for this analysis since the exact sites of phosphorylation were defined. After clustering and parameter refinement, 216 distinct clustering sets were obtained to create an ensemble of clustering sets. To identify candidate downstream components specific to DDR2 signalling, co-occurrence analysis was performed to establish the frequency at which pairs of phosphosites co-cluster robustly together in the ensemble of clustering sets. These data are represented by the co-occurrence matrix (Figure 3A). This matrix was then clustered to find groups of robustly co-clustered phosphoproteins to produce a final hard partition based on the ensemble results. This analysis was done using Hierarchical clustering and Ward linkage to produce 40 clusters. We found that the first seven clusters have a higher than average co-occurrence frequency and that the majority of DDR2 phosphosites are found in clusters 1–3 in the co-occurrence matrix (Figure 3B, and Supplementary Table S5 at http://www.biochemj.org/bj/454/bj4540501add.htm). These phosphorylation sites included DDR2 Tyr^740^ in cluster 1, Tyr^736^, Tyr^736^+Tyr^740^ and Tyr^684^ in cluster 2, and Tyr^813^ in cluster 3. Phosphorylated proteins in these clusters exhibit low phosphorylation levels at the early time points that reached a maximum at 24 h after collagen stimulation (Figure 3C). MCAM analysis highlights that despite these DDR2 phosphorylation sites having relatively similar dynamics, particular features of the individual sites can be distinguished. For example, the Tyr^740^ cluster is probably distinguished, by clustering, from the Tyr^813^ site by lower phosphorylation at early time points. Importantly, although there is a large degree of overlap between the DDR2-containing clusters (clusters 1–3), these clusters are distinct from the remainder of the dataset. Consistent with the role of DDR2 as an RTK, a plot of the individual pairwise co-occurrence values for the members in each cluster reveals that DDR2 phosphorylation sites robustly co-cluster with other tyrosine phosphosites in the dataset (Figure 3D).

**Figure 4:**
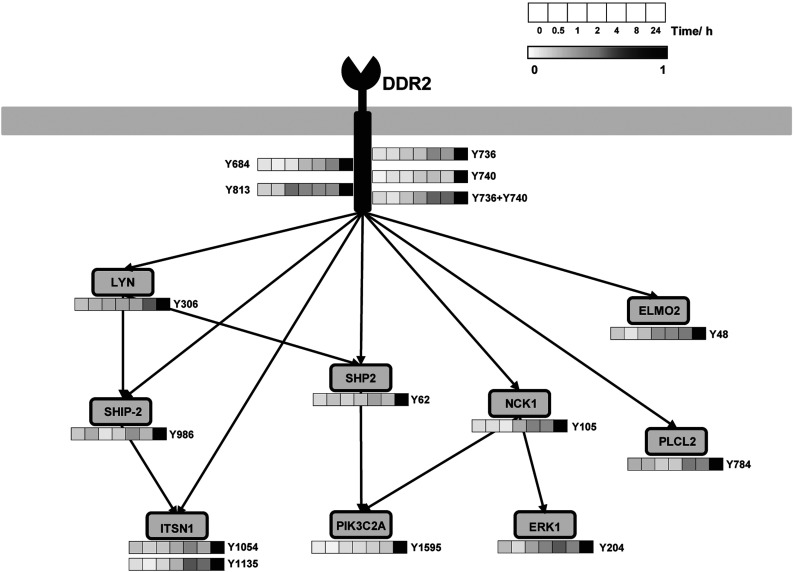
Visualization of the fold change in phosphorylation levels of important signal transduction adaptors and effector proteins upon temporal activation of DDR2 as determined by MCAM analysis Representative proteins were chosen from the first three clusters identified to co-cluster with high frequency with DDR2 receptor phosphorylation in the co-occurrence matrix analysis.

Robust co-clustering of DDR2 receptor phosphorylation with a large number of tyrosine-phosphorylated proteins in the dataset suggests that these proteins are likely to be candidate downstream components specific to the DDR2 signalling network. For instance, NCK1 is an adaptor protein that was found in cluster 2 and has previously been associated with DDR signalling [41]. Other members in these three clusters include important integrators of signal transduction, such as LYN, PIK3C2A, SHP-2, SHIP-2, ERK1 (extracellular-signal-regulated kinase 1) and PLCL2 (summarized in Figure 4). Acid control treatment of HEK-293-DDR2 cells did not lead to phosphorylation of DDR2 and its downstream components SHP-2, NCK1, LYN, PLCL2 and SHIP-2 as measured by ELISA (Supplementary Figure S2 at http://www.biochemj.org/bj/454/bj4540501add.htm), indicating that the observed increases in downstream protein phosphorylation are the result of collagen addition.

Collagen stimulation results in the activation of both the integrins and DDR2. The activation of these two distinct classes of receptors may occur on different timescales. To establish that these candidate proteins are specific to DDR2 and not due to integrin activation, HEK-293-EV (empty vector) control cells which express endogenous levels of collagen-binding integrins, but not DDR2 [25], were subjected to collagen treatment at both early (30 min and 2 h) and late (24 h) time points. As shown in Supplementary Figure S2, these cells fail to display increases in the phosphorylation of SHP-2, NCK1, LYN and PLCL2 at all time points, providing support that integrin activation is not the driver of the observed increases in tyrosine phosphorylation. The exception is SHIP-2, which has a small phosphorylation increase at 24 h in the HEK-293-EV cells, but a greater enhancement in the DDR2-expressing cells. Finally, to ascertain if activation of both DDR2 and integrins is required for signalling network propagation, HEK-293-DDR2 cells were pre-treated with a β1-integrin-blocking antibody (P5D2) before stimulation with collagen for 24 h (Supplementary Figure S3 at http://www.biochemj.org/bj/454/bj4540501add.htm). Blocking integrin activation does not alter the tyrosine phosphorylation status of downstream signalling compared with control. These experiments demonstrate that the observed up-regulation of protein tyrosine phosphorylation identified by the MCAM analysis occurs independently of integrin activation and is the result of DDR2 activation by collagen.

### SHP-2 tyrosine phosphorylation is dependent on DDR2 kinase activity

**Figure 5:**
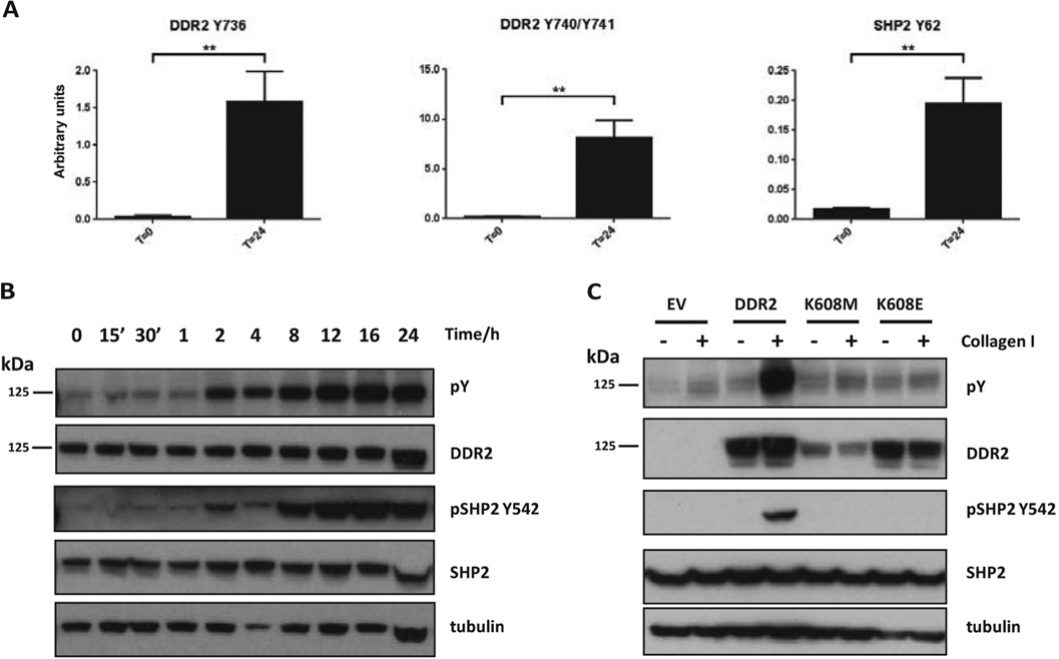
DDR2 phosphorylates SHP-2 in a temporal and kinase-dependent manner (**A**) SRM analysis of DDR2 activation loop (Tyr^736^ and Tyr^740^/Tyr^741^) and SHP-2 (Tyr^62^) phosphorylation at 0 and 24 h post-stimulation with collagen I (*n*=3). Values are means±S.E.M., **P<0.01 using paired Student's *t* test. Representative transitions are shown as indicated in Supplementary Table S3 (at http://www.biochemj.org/bj/454/bj4540501add.htm). (**B**) Immunoblot of DDR2 and SHP-2 (Tyr^542^) phosphorylation shows a temporal up-regulation of SHP-2 phosphorylation upon simulation with collagen. Phosphotyrosine (pY) is measured by the 4G10 antibody. (**C**) Immunoblot of kinase-dead DDR2 mutants (K608M and K608E) indicate that tyrosine phosphorylation of SHP-2 (Tyr^542^) is dependent on DDR2 kinase activity. EV, empty vector control.

Phosphorylation of SHP-2 at Tyr^62^, a protein tyrosine phosphatase that has previously been shown to be an important regulator of growth factor RTK signalling, was found to co-cluster robustly with DDR2 Tyr^740^ (90%), and with Tyr^684^ and Tyr^736^ (70%) (see Supplementary Table S6 at http://www.biochemj.org/bj/454/bj4540501add.htm for co-clustering frequency). We independently confirmed this finding by performing SRM analysis of this phosphorylation site on SHP-2 upon stimulation of HEK-293-DDR2 cells with collagen. By spiking in a synthetic heavy phosphopeptide of SHP-2 Tyr^62^ in a targeted proteomics experiment, we show that SHP-2 is robustly tyrosine phosphorylated 24 h after collagen stimulation (Figure 5A). This observation was further validated using a phospho-specific antibody for SHP-2 Tyr^542^, a phosphorylation site that is important for normal ERK activation in response to growth factor signalling [42]. In agreement with the phosphoproteomic data, temporal stimulation of HEK-293-DDR2 cells with collagen I showed an increase in receptor phosphorylation, accompanied by a concomitant increase in SHP-2 Tyr^542^ phosphorylation levels (Figure 5B). Correlation analysis of the phosphorylation levels of Tyr^62^ and Tyr^542^ on SHP-2 across 15 different measurements over a range of collagen stimulation time points by SRM and ELISA respectively (Supplementary Table S7 at http://www.biochemj.org/bj/454/bj4540501add.htm) demonstrates that there is a strong correlation between these two phosphorylation sites (Spearman correlation coefficient *r*=0.9321, *P*<0.0001). Importantly, the total abundance of the SHP-2 protein does not vary after 24 h exposure to collagen and thus protein abundance changes are not a contributing factor to the phosphorylation changes observed. SHP-2 tyrosine phosphorylation was dependent on DDR2 kinase activity, as HEK-293 cells expressing either control vector or a kinase-dead version of DDR2 (either K608M or K608E mutations) were unable to phosphorylate SHP-2 when cells were exposed to collagen I (Figure 5C). Taken together, these data indicate that SHP-2 is a downstream effector of DDR2 activation.

### SHP-2 is differentially tyrosine phosphorylated downstream of a subset of lung SCC-associated DDR2 mutants

**Figure 6:**
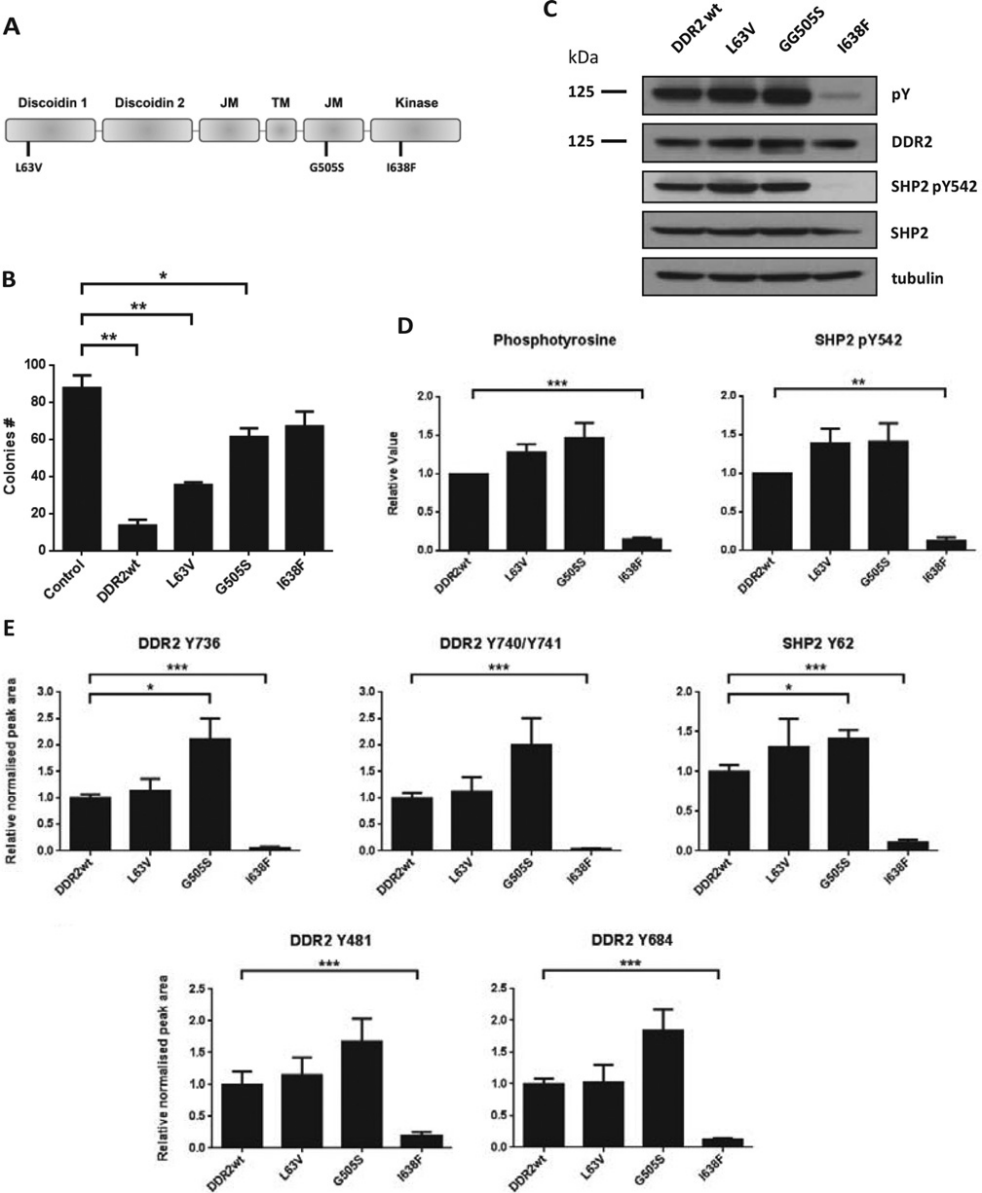
Targeted proteomic profiling of lung SCC DDR2 mutants (**A**) Domain organization and location of DDR2 point mutations used in the present study. JM, juxtamembrane; TM, transmembrane domain. (**B**) Colony formation assay of DDR2 mutants grown in 3D collagen I gels (*n*=3). Values are means±S.E.M., statistical significance of wild-type and mutant DDR2 data compared with the empty vector control was performed by ANOVA with Dunnett's post-test where **P<0.01 and *P<0.05. (**C**) Immunoblot of mutant DDR2 cells after stimulation with collagen I for 24 h. Phosphotyrosine (pY) is measured by the pY100 antibody. (**D**) Normalized densitometry measurements of phosphotyrosine and phosphorylated SHP-2 blots (*n*=3). ***P<0.001 and **P<0.01. Normalization is performed relative to the loading control, tubulin. (**E**) SRM analysis of DDR2 receptor and SHP-2 phosphorylation in wild-type and lung SCC point mutations post activation with collagen I at 24 h (*n*=3). Values are means±S.E.M., statistical significance of mutant DDR2 phosphorylation data compared with wild-type DDR2 was performed by paired Student's *t* test where ***P<0.001 and *P<0.05. Representative transitions are shown as indicated in Supplementary Table S3 at http://www.biochemj.org/bj/454/bj4540501add.htm.

A number of cancer-associated DDR2 point mutations were recently identified at low frequency through cancer genome sequencing studies in lung SCC [21]. These mutations are found throughout the *DDR2* gene and range from extracellular mutations to mutations in the KD [43]. The signalling pathways activated by these mutant receptors have not previously been characterized and we sought to determine whether SHP-2 was similarly phosphorylated downstream of these DDR2 mutants. HEK-293 cells stably expressing three mutants that are found in the extracellular collagen-binding discoidin domain (L63V), the juxtamembrane region (G505S) or the KD (I638F) were engineered (Figure 6A). In the original study by Hammerman et al. [21], the authors demonstrated in NIH 3T3 cells and BaF3 cells that a subset of DDR2 mutants, including L63V and I638F, are oncogenic. However, these assays were performed in the absence of collagen and the authors did not provide any experimental evidence that DDR2 was activated and phosphorylated in the cell lines tested. Activation of DDR2 by fibrillar collagen has previously been shown to inhibit proliferation of human melanoma and fibrosarcoma cells in 2D and 3D assays [44]. To determine the phenotypic effect of DDR2 and its lung cancer mutants in HEK-293 cells in the context of its natural ligand, collagen, a colony formation assay measuring the clonogenicity of these cells in 3D collagen I gels was performed as described previously [45–47]. Consistent with the tumour-suppressive role of DDR2 in cancer cells induced by fibrillar collagen [44], we found that wild-type DDR2 expression greatly reduced colony formation compared with the control cells (Figure 6B). Interestingly, DDR2 mutants showed variable responses to collagen I exposure, with the L63V and G505S showing intermediate suppression, and the I638F mutant displaying no significant effect on colony numbers compared with the control (Figure 6B).

Western blot analysis of these cells confirmed that the L63V and G505S mutant receptors were tyrosine-phosphorylated in a similar fashion to wild-type DDR2, whereas the I638F mutant showed a significant reduction in receptor phosphorylation (Figures 6C and 6D). We further demonstrate that L63V and G505S were able to phosphorylate SHP-2 at Tyr^542^, but I638F was incapable of doing so. To characterize the differences in DDR2 and SHP-2 tyrosine phosphorylation with site-specific resolution, wild-type and mutant cells were stimulated with collagen I and subjected to targeted SRM analysis. MS analysis revealed that distinct sites on DDR2 (Tyr^481^, Tyr^684^, Tyr^736^ and Tyr^740^/Tyr^741^) and SHP-2 (Tyr^62^) displayed varied phosphorylation responses among the different mutants (Figure 6E). A common observation across all of the phosphorylation sites is that the I638F mutant significantly diminished tyrosine phosphorylation on DDR2 and SHP-2 in agreement with the Western blot analysis.

## DISCUSSION

In the present study, we have performed an unbiased phosphoproteomic analysis of collagen receptor activation. Much of the published work on DDR2 has thus far focused on its extracellular domain and the nature of its binding to collagen [2,43]. The present study is, to our knowledge, the first comprehensive temporal study of collagen receptor signalling networks in cells. We have identified 424 phosphorylated proteins, including five tyrosine sites on the DDR2 receptor. Quantitative analysis of DDR2 receptor phosphorylation dynamics reveals that these sites are differentially regulated. The sites on the activation loop (Tyr^736^ and Tyr^740^) were not phosphorylated in the absence of collagen and showed delayed phosphorylation dynamics when stimulated, culminating in a sharp increase in phosphorylation levels from 8 to 24 h after collagen engagement. Also phosphorylated in a similar fashion are two novel receptor sites (Tyr^684^ and Tyr^813^) in the KD. Additionally, these sites were not identified in the *in vitro* kinase assay (Table 1), which suggests that they may be phosphorylated by kinases other than Src or DDR2 autophosphorylation. In contrast, Tyr^481^ on the juxtamembrane domain of the receptor displays constitutive phosphorylation which was confirmed *in vitro* with recombinant DDR2 which was phosphorylated even in its basal state. Owing to the lack of specific reagents (e.g. antibodies) targeted against these phosphorylation sites on DDR2, the present study represents the first demonstration that individual sites on DDR2 are regulated in a distinct temporal fashion, which may have consequences on the recruitment of downstream effectors and ultimately cellular behaviour.

MCAM analysis of the phosphoproteomic data identified a number of tyrosine-phosphorylated proteins that clustered robustly with DDR2 receptor phosphorylation (Supplementary Tables S5 and S6). Since collagen is known to stimulate an array of different receptors [2], this clustering approach was particularly useful for identifying proteins that are candidate downstream effectors of DDR2 signalling. Several of these effector proteins have been associated with DDR2 function. For instance, PI3K (phosphoinositide 3-kinase) and ERK activation is required for DDR2-dependent IL (interleukin)-6 secretion in primary human chondrocytes [48]. In addition, DDR2 increases the expression of matrix metalloproteinase-13 in synovial fibroblasts via the ERK pathway [49]. We focused our validation efforts on SHP-2, a protein tyrosine phosphatase that has previously been implicated in DDR1 signalling in MDCK (Madin–Darby canine kidney) cells [50]. This DDR1 study did not determine the tyrosine phosphorylation status of SHP-2 upon collagen stimulation. Using SRM and Western blot analysis, we demonstrate that SHP-2 is tyrosine-phosphorylated at two sites (Tyr^62^ and Tyr^542^) upon DDR2 activation. We further show that DDR2 kinase activity is required for SHP-2 phosphorylation, providing additional support that SHP-2 is a specific downstream effector of DDR2. It should be noted that the clustering analysis that we have performed is correlative in nature and serves to highlight the candidate protein phosphorylation sites that are co-regulated with DDR2 receptor phosphorylation. Although such clustering approaches are useful tools for hypothesis generation, whether these co-regulated events represent direct interactions between specific DDR2 phosphotyrosine sites and downstream candidate proteins requires additional experimental validation, such as the generation of tyrosine-to-phenylalanine mutants of DDR2 to probe for site-specific effects on SHP-2 phosphorylation.

We have recently shown that the collagen-binding integrins are active in the HEK-293-DDR2 cells used in the present study [25]. However, as we did not observe any phosphorylation sites on the collagen-binding integrin subunits in the phosphoproteomic dataset, we are unable to definitively identify the signalling events attributed to integrin activation using MCAM. A previous global phosphoproteomic analysis of integrin-mediated adhesion of HeLa cells identified 517 phosphorylation sites that were regulated within 2 h of adhesion on collagen-I-coated surfaces [51]. Two of these tyrosine phosphorylation sites [CDK1 (cyclin-dependent kinase 1) Tyr^15^ and DYRK1A (dual-specificity tyrosine-phosphorylation-regulated kinase 1A) Tyr^321^] were also found in our dataset, but displayed very different profiles from the integrin study. In the integrin study, CDK1 Tyr^15^ showed increasing phosphorylation levels reaching a maximum at 2 h after collagen stimulation, whereas DYRK1A Tyr^321^ displayed high basal phosphorylation levels that steadily decreased over time. Both of these sites showed constitutive phosphorylation levels that did not vary with time in our dataset. These differences could be the result of the distinct cell lines used in the two studies, the use of soluble collagen compared with collagen-coated surfaces, or the influence of DDR2 expression on integrin activation.

Unlike lung adenocarcinomas, SCCs of the lung are poorly characterized and there are currently no effective targeted therapies to treat this disease. The identification of point mutations in the *DDR2* gene in lung SCC by Hammerman et al. [21] suggests that this RTK may be a novel oncogenic target for therapy. However, their study was performed using classical transformation assays including anchorage dependence of NIH 3T3 cells grown in soft agar and IL-3-independent growth of BaF3 cells. In contrast, results from the present study demonstrate that HEK-293 cells expressing DDR2 have reduced clonogenic potential compared with control cells when grown in 3D collagen gels. Inhibition of cancer cell growth by fibrillar collagen is well documented and our results are consistent with published data indicating that DDR2 inhibits cancer cell proliferation when challenged with fibrillar collagen [44,52,53]. Loss of DDR2 has also been shown to predispose hepatic tissues to colon carcinoma cell growth and metastasis [54]. Interestingly, the present study shows that the I638F KD mutant restores the growth potential of these cells to control levels. The tyrosine phosphorylation status of the DDR2 mutants has not previously been established. We employ targeted proteomics to determine, with site-specific resolution, the phosphorylation profiles of DDR2 and SHP-2 in mutant-expressing cells. We find that the L63V and G505S mutants are signalling-competent and are capable of driving both receptor and SHP-2 tyrosine phosphorylation. The I638F KD mutant conversely displays a reduced ability to mediate DDR2 and SHP-2 phosphorylation. Taken together, our data suggests that in the presence of its natural ligand, DDR2 acts as a growth suppressor, and one potential mechanism of action of the I638F KD mutant is to alleviate this suppression by eliminating receptor and SHP-2 phosphorylation (Figures 6B and 6D). However, the relationship between growth suppression and signal transduction is likely to be complex and multivariate in nature since the G505S mutant displays high receptor and SHP-2 phosphorylation, but only has a small effect on growth suppression. The contrasting oncogenic and tumour-suppressive properties of DDR2 are likely to be context-dependent. The lung is an important site of collagen deposition and modification (such as by cross-linking) during tumour progression and the tumour-suppressive properties of DDR2 may be more pertinent in this context [55,56].

In conclusion, the results of the present study highlight the use of phosphoproteomics in combination with clustering techniques to establish a high-resolution map of DDR2 signalling effectors. We further demonstrate that SHP-2 is a major downstream component of DDR2 signalling and is phosphorylated in a subset of DDR2 lung SCC mutants. These findings provide an insight into the nature of DDR2 networks and signalling modulations that occur upon acquisition of cancer-associated mutations. We have previously shown that distinct oncogenic mutations in EGFR deploy differential pathway utilization in cancer cells, which has therapeutic implications in the clinic [26,27,57]. The results of the present study provide further support for this concept where the I638F KD mutant down-regulates receptor and SHP-2 phosphorylation, and alleviates DDR2-mediated growth suppression. Six kinase doman mutations have been identified in lung SCC, and three additional mutations in endometrial carcinoma and colorectal cancer [21]. Our findings suggest that restoring DDR2 receptor activity and its downstream signalling effectors, such as SHP-2, may be important for preserving tumour suppression in a subset of cancer-associated KD mutants.

## Online data

Supplementary data

Table S2
