# Supplementary material for: Phosphoproteomics of collagen receptor networks reveals SHP-2 phosphorylation downstream of wild-type DDR2 and its lung cancer mutants
Source: Biochem J. 2013 Aug 29;454(Pt 3):501–13. doi: 10.1042/BJ20121750 (PMC3893797; doi:10.1042/BJ20121750)
Supplement: Supplementary data [file bj4540501add.pdf]

BJ Signal

Biochem. J. (2013) **454**, 501–513 (Printed in Great Britain) doi:10.1042/BJ20121750

online data

SUPPLEMENTARY ONLINE DATA

Phosphoproteomics of collagen receptor networks reveals SHP-2 phosphorylation downstream of wild-type DDR2 and its lung cancer mutants

Leo K. IWAI*[1], Leo S. PAYNE*[1], Maciej T. LUCZYNSKI*, Francis CHANG*, Huifang XU†, Ryan W. CLINTON‡, Angela PAUL§, Edward A. ESPOSITO‡, Scott GRIDLEY‡, Birgit LEITINGER†, Kristen M. NAEGLE|| and Paul H. HUANG*[2]

*Protein Networks Team, Division of Cancer Biology, Institute of Cancer Research, London SW3 6JB, U.K., †National Heart and Lung Institute, Imperial College London, London SW7 2AZ, U.K., ‡Blue Sky Biotech Inc., Worcester, MA 01605, U.S.A., §Cancer Research UK Tumour Cell Signalling Unit, Institute of Cancer Research, London SW3 6JB, U.K., and ||Department of Biomedical Engineering, Washington University in St. Louis, St. Louis, MO 63130, U.S.A.

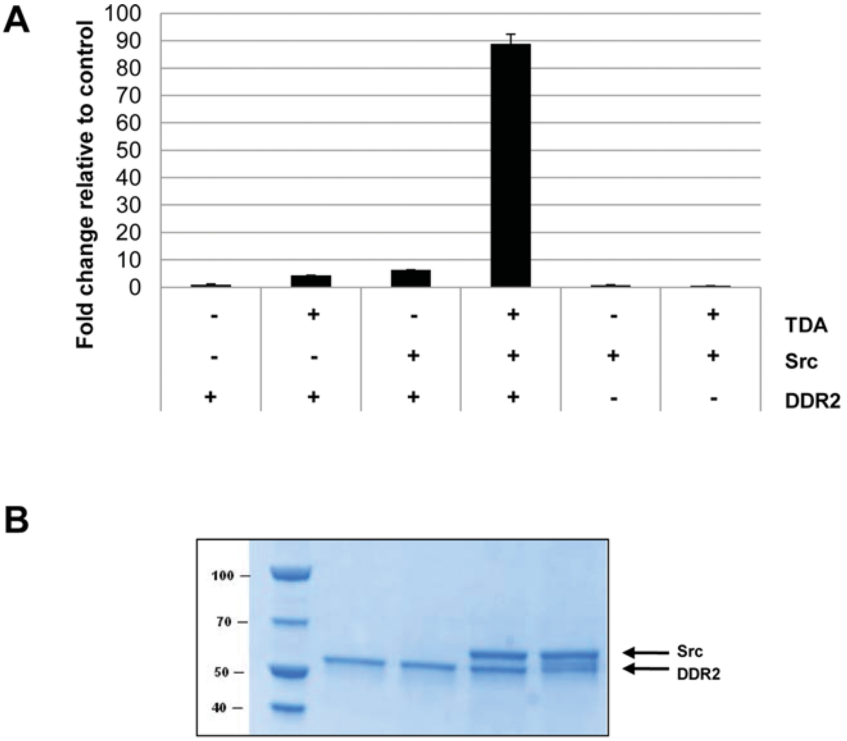

C

Enzyme: **DDR2 (R422-E855)**

MGGSHHHHHHHHHHSGENLYFQGASRQFWQKMLEKASRRMLDDEMTVSLSLPSDSSMFNNNRSSSPSEQGSNSTY
DRIFPLRPDYQEPSRLIRKLPEFAPGEEESGCSGVVKPVQPSGPEGVPHYAEADIVNLQGVTGGNTYSVPAVTMDLLS
GKDVAVEEFPRKLLTFKEKLGEGQFGEVHLCEVEGMEKFKDKDFALDVSANQPVLVAVKMLRADANKNARNDFLKEIKI
MSRLKDPNIIHLLAVCITDDPLCMITEYMENGDLNQFLSRHEPPNSSSSDVRTVSYTNLKFMATQIASGMKYLSSLNFVH
RDLATRNCLVGKNYTIKIADFGMSRNLYSGDYYRIQGRAVLPIRWMSWESILLGKFTTASDVWAFGVTLWETFTFCQEQ
PYSQLSDEQVIENTGEFFRDQGRQTYLPQPAICPDSVYKLMLSCWRRDTKNRPSFQEIHLLLLQQGDE

Enzyme: **Src**

MGGSHHHHHHHHHHSGENLYFQGASMGSNKSKPKDASQRRRSLEPAENVHGAGGGAFPASQTPSKPASADGHRG
PSAAFAPAAAEPKLFGGFNSSDTVTSPQRAGPLAGGVTTFVALYDYESRTETDLSFKKGERLQIVNNTEGDWWLAHS
LSTGQTGYIPSNYVAPSDSIQAEEWYFGKITRRESERLLLNAENPRGTFLVRESETTKGAYCLSVSDFDNAKGLNVKHY
KIRKLDSGGFYITSRTQFNSLQQLVAYYSKHADGLCHRLTTVCPTSKPQTQGLAKDAWEIPRESLRLEVKLGQGCFGE
VWMGTWNGTTRVAIKTLKPGTMSPEAFLQEAQVMKKLRHEKLVQLYAVVSEEPIYIVTEYMSKGSLLDFLKGETGKYL
RLPQLVDMAAQIASGMAYVERMNYVHRDLRAANILVGENLVCKVADFGLARLIEDNEYTARQGAKFPIKWTAPEAALY
GRFTIKSDVWSFGILLTELTTKGRVPYPGMVNREVLDQVERGYRMPCPPECPESLHDLMCQCWRKEPEERPTFEYLQ
AFLEDYFTSTEPQYQPGENL

Figure S1 Details of the DDR2 *in vitro* kinase assay

(**A**) *In vitro* kinase assay measuring the incorporation of ^{32}P into the Axltide substrate peptide. Src and DDR2 were mixed at a ratio of 1:20 together with TDA at a 1:10000 (TDA/enzyme) ratio in kinase assay buffer. Src does not phosphorylate the Axltide substrate peptide. (**B**) Coomassie Brilliant Blue-stained gel of *in vitro* kinase reaction samples that were subjected to LC-MS/MS analysis. (**C**) Sequence of recombinant DDR2 and Src that were used in the *in vitro* kinase assays.

[1] These authors contributed equally to this work.
[2] To whom correspondence should be addressed (email paul.huang@icr.ac.uk).

© 2013 The Author(s)
The author(s) has paid for this article to be freely available under the terms of the Creative Commons Attribution Licence (CC-BY) (http://creativecommons.org/licenses/by/3.0/) which permits unrestricted use, distribution and reproduction in any medium, provided the original work is properly cited.

Biochemical Journal www.biochemj.org

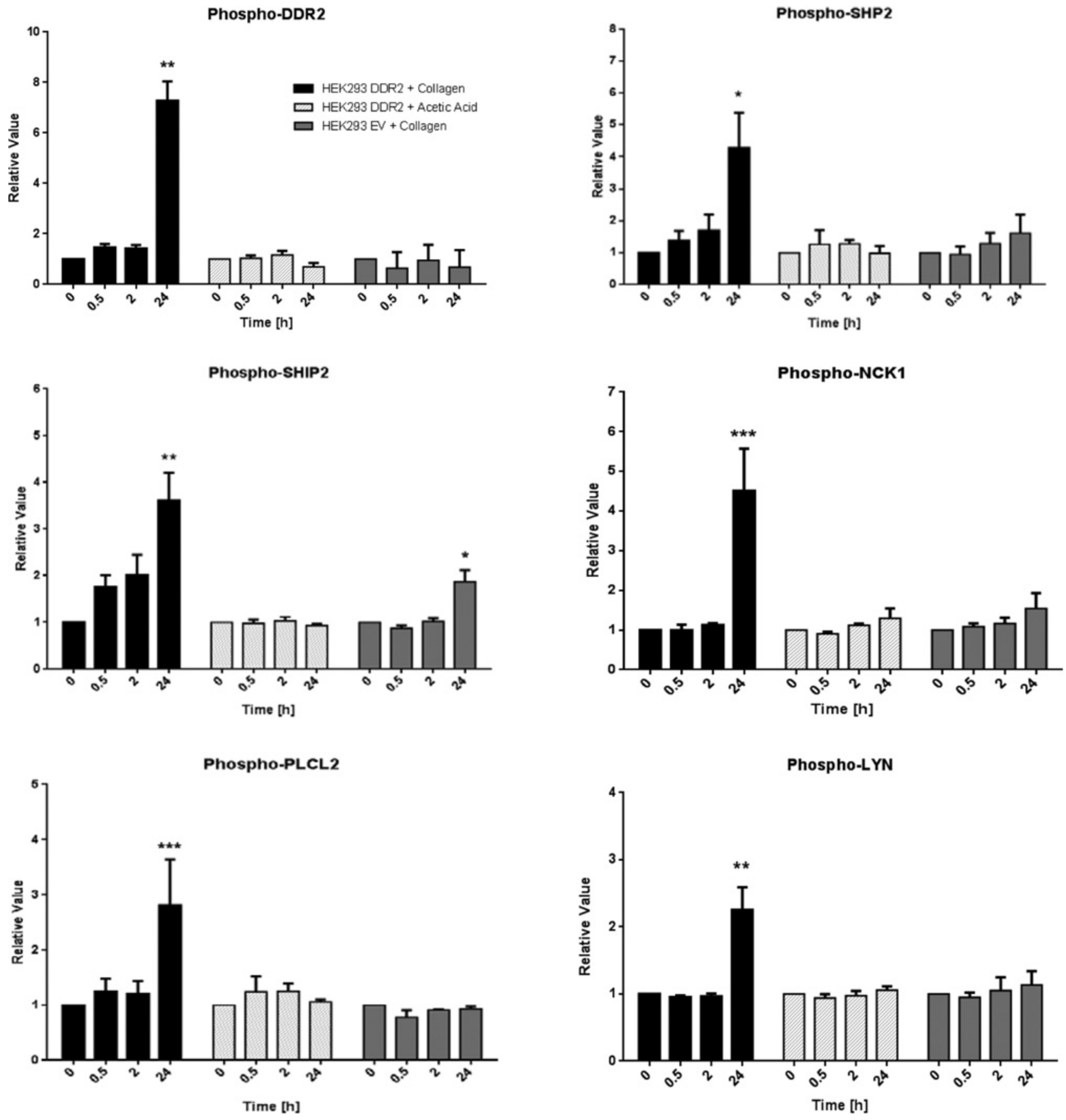

Figure S2 ELISA measurements of tyrosine phosphorylation levels of DDR2 and its downstream effectors (SHP2, SHIP-2, PLCL2, LYN and NCK1) in HEK-293-DDR2 cells at 0, 0.5, 2 and 24 h post collagen I stimulation ($n = 4$)

Values are means ± S.E.M. with $***P < 0.001$, $**P < 0.01$ and $*P < 0.05$, indicating a significant difference between $t = 0$ and $t = 24$ h as determined by paired Student's t test. As negative controls HEK-293-EV cells treated with collagen I and HEK-293-DDR2 cells treated with acetic acid were used.

© 2013 The Author(s)
The author(s) has paid for this article to be freely available under the terms of the Creative Commons Attribution Licence (CC-BY) (http://creativecommons.org/licenses/by/3.0/) which permits unrestricted use, distribution and reproduction in any medium, provided the original work is properly cited.

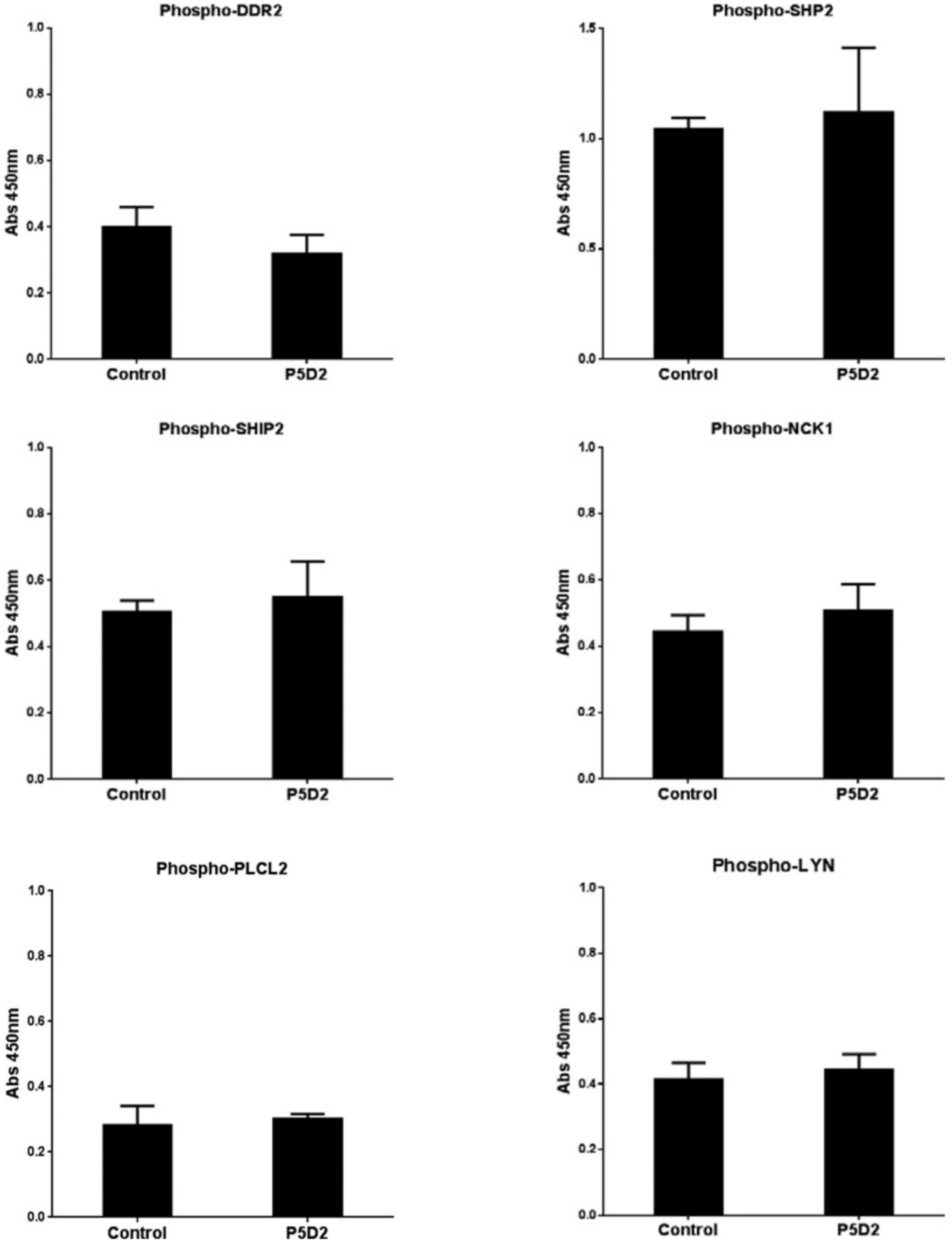

Figure S3 ELISA measurements of tyrosine phosphorylation levels of DDR2 and its downstream effectors (SHP2, SHIP-2, PLCL2, LYN and NCK1) in HEK-293-DDR2 at 24 h post collagen I stimulation ($n = 4$) in the presence or absence (control) of the P5D2 integrin β1-blocking antibody

Values are means $\pm$ S.E.M. There are no statistical differences between the P5D2 and control samples.

© 2013 The Author(s)
The author(s) has paid for this article to be freely available under the terms of the Creative Commons Attribution Licence (CC-BY) (http://creativecommons.org/licenses/by/3.0/) which permits unrestricted use, distribution and reproduction in any medium, provided the original work is properly cited.

Table S1 Primer sequences for generation of DDR2 mutants

The bases shown in bold indicate the nucleotide that was mutated from the corresponding wild-type to generate mutants using site-directed mutagenesis methodology.

Mutant	Direction	Primer sequence (5′→3′)
L63V	Forward	CTGCCAAATATGGAAGG**G**TGGACTCAGAAGAAGGG
	Reverse	CCCTTCTTCTGAGTCCA**C**CCTTCCATATTTGGCAG
G505S	Forward	GAGTCAGGCTGCAGC**A**GTGTTGTGAAGCCAG
	Reverse	CTGGCTTCACAACAC**T**GCTGCAGCCTGACTC
K608M	Forward	GTCCTGGTGGCTGTGA**TG**ATGCTCCGAGCAGATG
	Reverse	CATCTGCTCGGAGCAT**CA**TCACAGCCACCAGGAC
K608E	Forward	TGTCCTGGTGGCTGTG**G**A**G**ATGCTCCGAGCAGATG
	Reverse	CATCTGCTCGGAGCAT**C**T**C**CACAGCCACCAGGACA
I638F	Forward	CTCAAGGACCCAAACATC**T**TCCATCTATTAGCTGTGT
	Reverse	ACACAGCTAATAGATGGA**A**GATGTTTGGGTCCTTGAG

Table S2 Heavy phosphopeptide sequences used in the SRM assay

Phosphosite	Peptide	Amount spiked per sample
DDR2 Tyr^{481}	IFPLRPD[Tyr(PO_3H_2)]QEPS[Arg($^{13}C_6$;$^{15}N_4$)]	500 fmol
DDR2 Tyr^{684}	TVS[Tyr(PO_3H_2)]TNL[Lys($^{13}C_6$;$^{15}N_2$)]	100 fmol
DDR2 Tyr^{736}	NL[Tyr(PO_3H_2)]SGDYY[Arg($^{13}C_6$;$^{15}N_4$)]	100 fmol
DDR2 Tyr^{740}	NLYSGD[Tyr(PO_3H_2)]Y[Arg($^{13}C_6$;$^{15}N_4$)]	200 fmol
SHP2 Tyr^{62}	IQNTGD[Tyr(PO_3H_2)]YDLYGGE[Lys($^{13}C_6$;$^{15}N_2$)]	2.5 pmol

Table S3 Transitions used for the SRM assay

Values in bold indicate representative transitions used in the Figures.

Phosphosite	Peptide	Transition (Q1/Q3)	Fragment ion ID	Collision energy (V)
DDR2 Tyr^{481} (heavy)	IFPLRPD[Tyr(PO_3H_2)]QEPS[Arg(^{13}C6;^{15}N4)]	**569.9/369.2**	Y3	36.6
		569.9/869.3	Y6	36.6
		569.9/498.3	Y4	36.6
DDR2 Tyr^{684} (heavy)	TVS[Tyr(PO_3H_2)]TNL[Lys($^{13}C_6$;$^{15}N_2$)]	**507.2/813.3**	Y6	28.7
		507.2/201.1	B2	28.7
		507.2/726.3	Y5	28.7
		507.2/483.3	Y4	28.7
DDR2 Tyr^{736} (heavy)	NL[Tyr(PO_3H_2)]SGDYY[Arg($^{13}C_6$;$^{15}N_4$)]	620.7/1013.3	Y7	35.0
		**620.7/770.3**	Y6	35.0
		620.7/511.3	Y3	35.0
		620.7/683.3	Y5	35.0
DDR2 Tyr^{740} (heavy)	NLYSGD[Tyr(PO_3H_2)]Y[Arg($^{13}C_6$;$^{15}N_4$)]	620.7/1013.3	Y7	35.0
		**620.7/391.2**	B3	35.0
		620.7/591.1	Y3	35.0
		620.7/706.2	Y4	35.0
SHP2 Tyr^{62} (heavy)	IQNTGD[Tyr(PO_3H_2)]YDLYGGE[Lys($^{13}C_6$;$^{15}N_2$)]	912.4/789.4	Y7	53.7
		912.4/674.4	Y6	53.7
		912.4/561.3	Y5	53.7
		**912.4/398.2**	Y4	53.7
DDR2 Tyr^{481} (endogenous)	IFPLRPD[Tyr(PO_3H_2)]QEPSR	**566.6/359.2**	Y3	36.6
		566.6/859.3	Y6	36.6
		566.6/488.3	Y4	36.6
DDR2 Tyr^{684} (endogenous)	TVS[Tyr(PO_3H_2)]TNLK	**503.2/805.3**	Y6	28.7
		503.2/201.1	B2	28.7
		503.2/718.3	Y5	28.7
		503.2/475.3	Y4	28.7
DDR2 Tyr^{736} (endogenous)	NL[Tyr(PO_3H_2)]SGDYYR	615.7/1003.3	Y7	35.0
		**615.7/760.3**	Y6	35.0
		615.7/501.2	Y3	35.0
		615.7/673.3	Y5	35.0
DDR2 Tyr^{740} (endogenous)	NLYSGD[Tyr(PO_3H_2)]YR	615.7/1003.3	Y7	35.0
		**615.7/391.2**	B3	35.0
		615.7/581.1	Y3	35.0
		615.7/696.2	Y4	35.0
SHP2 Tyr^{62} (endogenous)	IQNTGD[Tyr(PO_3H_2)]YDLYGGEK	908.4/781.37	Y7	53.7
		908.4/666.4	Y6	53.7
		908.4/553.3	Y5	53.7
		**908.4/390.2**	Y4	53.7

© 2013 The Author(s)
The author(s) has paid for this article to be freely available under the terms of the Creative Commons Attribution Licence (CC-BY) (http://creativecommons.org/licenses/by/3.0/) which permits unrestricted use, distribution and reproduction in any medium, provided the original work is properly cited.

Table S4 Dataset preparation and MCAM implementation

Parameters used for the initial MCAM analysis and pruned from the final round of feature selection. FFT, fast Fourier transform.

Parameter	Original parameters of clustering	Removed during feature selection
K	5, 8, 11, 14, 17, 20, 23, 26, 29	5
Transform	Centre, FFT, differential, no transform, z-score, normMax, rangeScale, square root, pareto	Centre, FFT, differential
Distance	Correlation, Euclidean, cityblock, cosine, Chebychev	Correlation
Algorithm	Hierarchical, k-means, affinity propagation, self-organizing maps	Hierarchical
Number of cluster sets	950	216 (remaining)

Table S5 Phosphorylation sites in the top seven clusters in MCAM analysis

The sites in bold highlight the SHIP-2 and DDR2 phosphorylation sites in each cluster.

Cluster 1	Cluster 2	Cluster 3	Cluster 4	Cluster 5	Cluster 6	Cluster 7
PFDN6 Tyr^{82}	SF3B14 Tyr^{86}	BAIAP2 Tyr^{491}	EDC4 Ser^{723}	MYL9 Thr^{19}	AAK1 Ser^{637}	PRKAR1A Ser^{83}
BANF1 Tyr^{43}	**DDR2 Tyr^{684}**	SCRIB Tyr^{1360}	SFRS15 Ser^{154}	PYGL Ser^{15}	ZC3H13 Ser^{64}	TPR Ser^{2155}
SHIP-2 Tyr^{986}	SDCBP Tyr^{50}	SNX9 Tyr^{269}	NOC2L Ser^{672}, Ser^{673}	SHIP-2 Tyr^{1135}	SRRM2 Ser^{1987}	SRRM2 Ser^{2100}, Thr^{2104}
UTRN Tyr^{3111}	EPS15L1 Tyr^{74}	TUBB Tyr^{340}	CCDC88A Tyr^{1799}	HSPA1A Tyr^{611}	SRRM2 Thr^{1208}	TP53BP1 Ser^{380}
MAGOH Tyr^{123}	**DDR2 Tyr^{736}**	RNPS1 Tyr^{205}	MAPK14 Tyr^{182}	AKT1S1 Ser^{183}	SRRM2 Ser^{1694}	MAP1S Ser^{657}
**SHP2 Tyr^{62}**	ITSN1 Tyr^{1132}	IGF2BP2 Tyr^{40}	TBC1D15 Ser^{227}	TRIM28 Tyr^{517}	RPLP1 Ser^{101}, Ser^{104}	SRRM2 Ser^{2272}
PSAT1 Tyr^{346}	NCK1 Tyr^{112}	DDR1 Tyr^{796}	CLPB Ser^{23}	PTPRA Tyr^{798}	KLC2 Ser^{581}	TNKS1BP1 Ser^{691}
LCP1 Tyr^{28}	IGF2BP3 Tyr^{39}	ERK1 Tyr^{204}	RPLP1 Ser^{101}	SYK Tyr^{323}	SPTAN1 Ser^{1217}	SRRM2 Thr^{1492}
PIN4 Tyr^{122}	PLCL2 Tyr^{784}	VIM Tyr^{117}	CRKRS Ser^{685}, Ser^{681}			STMN1 Ser^{16}, Ser^{25}
EDC4 Ser^{729}	**DDR2 Tyr^{736}, Tyr^{740}**	LYN Tyr^{306}	JUN Ser^{73}			SRRM2 Ser^{1179}
**DDR2 Tyr^{740}**	AGFG1 Tyr^{327}	HSPA1A Tyr^{41}	SPAG9 Thr^{217}			
CLTC Tyr^{1487}	ELMO2 Tyr^{48}	LPP Tyr^{296}	SFRS1 Tyr^{189}			
SF3B14 Tyr^{61}	ANKRD39 Tyr^{65}	**DDR2 Tyr^{813}**				
FASN Tyr^{130}	ACBD3 Ser^{43}	PABPC1 Tyr^{54}				
ITSN1 Tyr^{1054}	ADD1 Tyr^{407}					
PIK3C2A Tyr^{1595}						
TRAP1 Tyr^{498}						
DDR1 Tyr^{792}						
GTF2E1 Tyr^{91}						
AK2 Tyr^{190}						
TUBGCP3 Tyr^{114}						
PSMC3 Tyr^{132}						
ACTB Tyr^{218}						

© 2013 The Author(s)
The author(s) has paid for this article to be freely available under the terms of the Creative Commons Attribution Licence (CC-BY) (http://creativecommons.org/licenses/by/3.0/) which permits unrestricted use, distribution and reproduction in any medium, provided the original work is properly cited.

© The Authors Journal compilation © 2013 Biochemical Society

Table S6 Co-occurrence frequency listed by the DDR2 phosphorylation sites

The sites in bold highlight SHP-2 Tyr^{62} phosphorylation and their respective co-occurrence frequency with specific DDR2 phosphorylation sites.

DDR2 Tyr^{481}

Phosphorylation site	Co-occurrence frequency
DDR2 Tyr^{481}	1.00
CRKL Tyr^{207}	0.67
TPI1 Ser^{21}	0.62
RANBP1 Ser^{60}	0.55
COIL Ser^{301}	0.51
WDR75 Ser^{796}	0.50

DDR2 Tyr^{684}

Phosphorylation site	Co-occurrence frequency
DDR2 Tyr^{684}	1.00
AGFG1 Tyr^{327}	0.91
NCK1 Tyr^{112}	0.90
DDR2 Tyr^{736}	0.86
IGF2BP3 Tyr^{39}	0.86
SDCBP Tyr^{50}	0.83
ANKRD39 Tyr^{65}	0.82
EPS15L1 Tyr^{74}	0.82
SF3B14 Tyr^{86}	0.81
ADD1 Tyr^{407}	0.81
ELMO2 Tyr^{48}	0.77
ITSN1 Tyr^{1132}	0.76
ACBD3 Ser^{43}	0.74
PSAT1 Tyr^{346}	0.74
ITSN1 Tyr^{1054}	0.73
CLTC Tyr^{1487}	0.71
SHIP-2 Tyr^{986}	0.70
TUBGCP3 Tyr^{114}	0.70
**SHP2 Tyr^{62}**	0.69
DDR2 Tyr^{736}, Tyr^{740}	0.69
TRAP1 Tyr^{498}	0.69
DDR1 Tyr^{792}	0.69
AK2 Tyr^{190}	0.69
PIN4 Tyr^{122}	0.69
SF3B14 Tyr^{61}	0.69
FASN Tyr^{130}	0.69
PIK3C2A Tyr^{1595}	0.68
GTF2E1 Tyr^{91}	0.68
PFDN6 Tyr^{82}	0.68
DDR2 Tyr^{740}	0.68
ACTB Tyr^{218}	0.67
UTRN Tyr^{3111}	0.67
LCP1 Tyr^{28}	0.66
BANF1 Tyr^{43}	0.65
PLCL2 Tyr^{784}	0.65
MAGOH Tyr^{123}	0.65
EDC4 Ser^{729}	0.65
PSMC3 Tyr^{132}	0.64
LYN Tyr^{306}	0.58
SNX9 Tyr^{269}	0.57
TUBB Tyr^{340}	0.56
RNPS1 Tyr^{205}	0.55
DDR1 Tyr^{796}	0.55
SCRIB Tyr^{1360}	0.53
ERK1 Tyr^{204}	0.53
VIM Tyr^{117}	0.50
DDR2 Tyr^{813}	0.50

DDR2 Tyr^{736}

Phosphorylation site	Co-occurrence frequency
DDR2 Tyr^{736}	1.00
SF3B14 Tyr^{86}	0.94
NCK1 Tyr^{112}	0.91
IGF2BP3 Tyr^{39}	0.91
SDCBP Tyr^{50}	0.87
DDR2 Tyr^{684}	0.86
AGFG1 Tyr^{327}	0.86
ADD1 Tyr^{407}	0.85
ITSN1 Tyr^{1054}	0.84
ANKRD39 Tyr^{65}	0.84
EPS15L1 Tyr^{74}	0.83
ACBD3 Ser^{43}	0.81
ITSN1 Tyr^{1132}	0.79
ELMO2 Tyr^{48}	0.79
PIN4 Tyr^{122}	0.78
PLCL2 Tyr^{784}	0.76
PSAT1 Tyr^{346}	0.73
AK2 Tyr^{190}	0.73
TUBGCP3 Tyr^{114}	0.72
DDR2 Tyr^{736}, Tyr^{740}	0.71
FASN Tyr^{130}	0.70
SHIP-2 Tyr^{986}	0.70
**SHP2 Tyr^{62}**	0.69
TRAP1 Tyr^{498}	0.69
CLTC Tyr^{1487}	0.68
SF3B14 Tyr^{61}	0.65
DDR1 Tyr^{792}	0.65
GTF2E1 Tyr^{91}	0.64
PFDN6 Tyr^{82}	0.64
UTRN Tyr^{3111}	0.64
DDR2 Tyr^{740}	0.64
PIK3C2A Tyr^{1595}	0.64
PSMC3 Tyr^{132}	0.63
ACTB Tyr^{218}	0.63
LYN Tyr^{306}	0.62
BANF1 Tyr^{43}	0.62
MAGOH Tyr^{123}	0.62
LCP1 Tyr^{28}	0.62
EDC4 Ser^{729}	0.61
DDR2 Tyr^{813}	0.57
SCRIB Tyr^{1360}	0.56
ERK1 Tyr^{204}	0.56
DDR1 Tyr^{796}	0.56
RNPS1 Tyr^{205}	0.55
SNX9 Tyr^{269}	0.54
TUBB Tyr^{340}	0.52
LPP Tyr^{296}	0.50

DDR2 Tyr^{740}

Phosphorylation site	Co-occurrence frequency
PFDN6 Tyr^{82}	1.00
DDR2 Tyr^{740}	1.00
PIK3C2A Tyr^{1595}	0.98
DDR1 Tyr^{792}	0.97
LCP1 Tyr^{28}	0.97
GTF2E1 Tyr^{91}	0.97
ACTB Tyr^{218}	0.97
BANF1 Tyr^{43}	0.95
EDC4 Ser^{729}	0.93
CLTC Tyr^{1487}	0.93
SF3B14 Tyr^{61}	0.92
MAGOH Tyr^{123}	0.92
UTRN Tyr^{3111}	0.90
**SHP2 Tyr^{62}**	0.90
TRAP1 Tyr^{498}	0.89
PSMC3 Tyr^{132}	0.88
TUBGCP3 Tyr^{114}	0.87
PSAT1 Tyr^{346}	0.85
SHIP-2 Tyr^{986}	0.85
AK2 Tyr^{190}	0.77
FASN Tyr^{130}	0.75
ITSN1 Tyr^{1054}	0.75
PIN4 Tyr^{122}	0.71
AGFG1 Tyr^{327}	0.70
DDR2 Tyr^{684}	0.68
PLCL2 Tyr^{784}	0.65
SF3B14 Tyr^{86}	0.64
DDR2 Tyr^{736}	0.64
ACBD3 Ser^{43}	0.63
IGF2BP3 Tyr^{39}	0.58
SDCBP Tyr^{50}	0.58
NCK1 Tyr^{112}	0.58
ANKRD39 Tyr^{65}	0.55
EPS15L1 Tyr^{74}	0.55
ADD1 Tyr^{407}	0.55
ITSN1 Tyr^{1132}	0.51
ELMO2 Tyr^{48}	0.51
JUN Ser^{73}	0.51
SFRS1 Tyr^{189}	0.50

DDR2 Tyr^{736}, Tyr^{740}

Phosphorylation site	Co-occurrence frequency
DDR2 Tyr^{736} Tyr^{740}	1.00
ITSN1 Tyr^{1132}	0.88
ELMO2 Tyr^{48}	0.88
EPS15L1 Tyr^{74}	0.83
ANKRD39 Tyr^{65}	0.83
RNPS1 Tyr^{205}	0.80
ADD1 Tyr^{407}	0.79
NCK1 Tyr^{112}	0.78
SCRIB Tyr^{1360}	0.78
IGF2BP3 Tyr^{39}	0.77
DDR1 Tyr^{796}	0.77
ERK1 Tyr^{204}	0.77
LYN Tyr^{306}	0.77
TUBB Tyr^{340}	0.74
SDCBP Tyr^{50}	0.74
SNX9 Tyr^{269}	0.73
LPP Tyr^{296}	0.72
DDR2 Tyr^{736}	0.71
SF3B14 Tyr^{86}	0.70
ACBD3 Ser^{43}	0.70
DDR2 Tyr^{684}	0.69
VIM Tyr^{117}	0.67
DDR2 Tyr^{813}	0.67
IGF2BP2 Tyr^{40}	0.65
AGFG1 Tyr^{327}	0.65
PABPC1 Tyr^{54}	0.63
PLCL2 Tyr^{784}	0.63
HSPA1A Tyr^{41}	0.62
ITSN1 Tyr^{1054}	0.59
PIN4 Tyr^{122}	0.59
BAIAP2 Tyr^{491}	0.56
AK2 Tyr^{190}	0.50

DDR2 Tyr^{813}

Phosphorylation site	Co-occurrence frequency
DDR2 Tyr^{813}	1.00
ERK1 Tyr^{204}	0.84
LYN Tyr^{306}	0.82
LPP Tyr^{296}	0.82
SCRIB Tyr^{1360}	0.81
RNPS1 Tyr^{205}	0.80
DDR1 Tyr^{796}	0.80
SNX9 Tyr^{269}	0.78
TUBB Tyr^{340}	0.77
BAIAP2 Tyr^{491}	0.73
IGF2BP2 Tyr^{40}	0.72
VIM Tyr^{117}	0.69
PABPC1 Tyr^{54}	0.68
DDR2 Tyr^{736}, Tyr^{740}	0.67
ELMO2 Tyr^{48}	0.64
HSPA1A Tyr^{41}	0.63
SDCBP Tyr^{50}	0.61
ITSN1 Tyr^{1132}	0.60
ADD1 Tyr^{407}	0.60
SF3B14 Tyr^{86}	0.59
IGF2BP3 Tyr^{39}	0.59
PLCL2 Tyr^{784}	0.59
EPS15L1 Tyr^{74}	0.58
ANKRD39 Tyr^{65}	0.58
DDR2 Tyr^{736}	0.57
NCK1 Tyr^{112}	0.57
JUN Ser^{73}	0.57
ACBD3 Ser^{43}	0.57
PIN4 Tyr^{122}	0.55
ITSN1 Tyr^{1054}	0.53
SFRS1 Tyr^{189}	0.53
SFRS15 Ser^{154}	0.52
CCDC88A Tyr^{1799}	0.52
SPAG9 Thr^{217}	0.52
DDR2 Tyr^{684}	0.50

© 2013 The Author(s)

The author(s) has paid for this article to be freely available under the terms of the Creative Commons Attribution Licence (CC-BY) (http://creativecommons.org/licenses/by/3.0/) which permits unrestricted use, distribution and reproduction in any medium, provided the original work is properly cited.

Table S7 Correlation analysis for phosphorylation of SHP-2 Tyr^{62} and Tyr^{542}

HEK-293-DDR2 cells were treated with 20 μg/ml collagen I and harvested across a range of different time points. For each time point, equivalent lysates were harvested for both SRM and ELISA experiments. Each sample represents a time point after collagen stimulation of cells. Data have been normalized to sample 15. Spearman correlation coefficient $r = 0.9321$, $P < 0.0001$.

Sample	ELISA (Tyr^{542})	SRM (Tyr^{62})
1	0.137	0.063
2	0.362	0.283
3	0.521	0.403
4	0.541	0.336
5	0.620	0.360
6	0.655	0.525
7	0.699	0.391
8	0.809	0.457
9	0.848	0.618
10	0.969	1.870
11	1.099	0.664
12	1.107	0.719
13	1.177	2.891
14	1.868	3.083
15	1.000	1.000

Received 21 November 2012/1 July 2013; accepted 4 July 2013
Published as BJ Immediate Publication 4 July 2013, doi:10.1042/BJ20121750

© 2013 The Author(s)
The author(s) has paid for this article to be freely available under the terms of the Creative Commons Attribution Licence (CC-BY) (http://creativecommons.org/licenses/by/3.0/) which permits unrestricted use, distribution and reproduction in any medium, provided the original work is properly cited.
